# Downregulation of DNA methylation enhances differentiation of THP-1 cells and induces M1 polarization of differentiated macrophages

**DOI:** 10.1038/s41598-023-40362-8

**Published:** 2023-08-12

**Authors:** Junyoung Park, Yongyang Luo, Jin Woo Park, Song Hyun Kim, Ye Joo Hong, Younghyun Lim, Young-Jin Seo, Jeehyeon Bae, Sang Beom Seo

**Affiliations:** 1 Department of Life Science, College of Natural Sciences, Chung-Ang University, Seoul, 06974 Republic of Korea; 2 College of Pharmacy, Chung-Ang University, Seoul, 06974 Republic of Korea

**Keywords:** Cancer, Molecular biology

## Abstract

DNA methylation is an epigenetic modification that regulates gene expression and plays an essential role in hematopoiesis. UHRF1 and DNMT1 are both crucial for regulating genome-wide maintenance of DNA methylation. Specifically, it is well known that hypermethylation is crucial characteristic of acute myeloid leukemia (AML). However, the mechanism underlying how DNA methylation regulates the differentiation of AML cells, including THP-1 is not fully elucidated. In this study, we report that UHRF1 or DNMT1 depletion enhances the phorbol-12-myristate-13-acetate (PMA)-induced differentiation of THP-1 cells. Transcriptome analysis and genome-wide methylation array results showed that depleting UHRF1 or DNMT1 induced changes that made THP-1 cells highly sensitive to PMA. Furthermore, knockdown of UHRF1 or DNMT1 impeded solid tumor formation in xenograft mouse model. These findings suggest that UHRF1 and DNMT1 play a pivotal role in regulating differentiation and proliferation of THP-1 cells and targeting these proteins may improve the efficiency of differentiation therapy in AML patients.

## Introduction

Accumulating evidence has shown that dysregulation of DNA methylation is a hallmark of acute myeloid leukemia (AML) initiation and progression. Overexpression of MLL-AF9 in human hematopoietic stem cells (HSCs) results in a distinct DNA methylation signature, suggesting a close correlation between MLL fusion proteins and aberrant DNA methylation in leukemic transformation^1^.

MLL-AF9-driven AML-type leukemic THP-1 cells, which derived from acute monocytic leukemia (AMoL) patient, differentiate into macrophages upon treatment with phorbol-12-myristate-13-acetate (PMA)^2–4^. Previous studies have suggested that inhibitors targeting MLL fusion proteins, such as DOT1L^5^, BRD4^6^, MOF^7^, and SIRT^8^ can function as potential therapeutic agents. In addition, co-treatment using all-trans retinoic acid (ATRA) and a histone deacetylase (HDAC) inhibitor has been used as an epigenetic therapy for AML, and it works by inducing differentiation through hyperacetylation of histones^9^.

Ubiquitin-like-containing PHD and RING finger domains protein 1 (UHRF1) is a master regulator in the maintenance of DNA methylation, as it recruits DNA (cytosine-5)-methyltransferase 1 (DNMT1) to newly synthesized DNA. The knockout of *UHRF1* in mouse embryonic stem cells results in global loss of DNA methylation^10,11^. The upregulation of UHRF1 in various cancers and promyelocytic leukemia^12^ and its importance in cancer progression have been investigated in many studies^13^. Previously, we found that UHRF1 is upregulated in leukemia and that UHRF1 acts in the AML cell line HL-60 and other cancers via G9a-mediated transcriptional regulation^14^. We also found that PMA treatment induces leukemia cell differentiation by recruiting transcriptional repressor protein YY1 and histone-lysine N-methyltransferase EHMT2 (G9a) and represses the transcription of *UHRF1* via histone H3 lysine 9 methylation (H3K9me)^14^. Interestingly, a previous study suggested that UHRF1 modulates the fate of HSCs by showing that the ablation of UHRF1 induces erythroid-biased differentiation of HSCs, leading to hematopoietic failure and lethality^15^.

DNMTs, including DNMT1, DNA (cytosine-5)-methyltransferase 3A and 3B (DNMT3A and DNMT3B), are major proteins regulating genome-wide DNA methylation. Specifically, DNMT1 regulates maintenance DNA methylation and DNMT3A and DNMT3B are involved in de novo DNA methylation. A previous in vivo study has shown that DNMT1 has a crucial role in MLL-AF9 leukemia development^16^. In addition, DNMT1 inhibition by specific inhibitors showed tumor regression and increased survival of AML mouse models^17^. Also, there is a clinical report that about 20% of AML patients have mutations in DNMT3A^18^. Another study has suggested that DNMT3B plays a tumor-suppressive role in MLL-AF9-driven AML progression^19^.

Hematopoietic differentiation is disrupted in leukemic cells owing to genetic or gene expression abnormalities^20,21^. Since proliferating leukemic cells lack the ability to function as normal immune cells, leukemia patients with high white blood cell counts have impaired immune functions^20^. Therefore, inducing leukemic cells to differentiate into normal cells could be a promising therapeutic approach for treating AML patients. However, cytotoxicity of differentiation-inducing agents is the major problem to overcome^22^.

In this study, we investigated the expression pattern and clinical relevance of DNA methylation-related genes, including UHRF1 and DNMTs, in patients with AML using different genome-wide profiling and public databases. We found that the expression of UHRF1 and DNMT1 dramatically decreased after THP-1 cell differentiation, and that depletion of UHRF1 or DNMT1 positively regulated the differentiation of THP-1 cells after treatment with PMA. By performing RNA sequencing, we found that UHRF1 and DNMT1 regulate the expression of genes related to THP-1 cell differentiation. Furthermore, we analyzed global DNA methylation patterns following depletion of UHRF1 or DNMTs using DNA methylation array and showed that the changes in DNA methylation patterns are related to the differentiation of THP-1 cells. These findings suggest that UHRF1 and DNMT1 play roles in regulating PMA-induced differentiation of THP-1 cells by regulating transcription and DNA methylation of differentiation-related genes.

## Results

### Transcriptome changes dynamically during THP-1 differentiation

**Figure 1:**
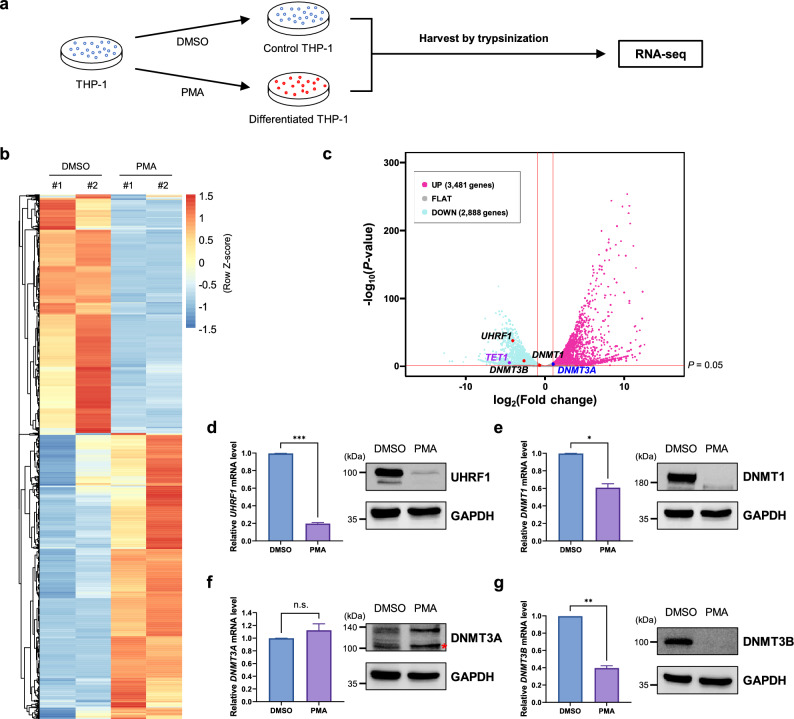
PMA treatment induces dynamic transcriptome variations in THP-1 cells. (**a**) Schematic image of sample preparation process for RNA-seq. Total RNA was extracted after 48 h of DMSO or PMA treatment. DMSO: dimethyl sulfoxide, PMA: phorbol-12-myristate-13-acetate. (**b**) Heatmap representing PMA-induced differentially expressed genes (DEGs) in THP-1 cells. (**c**) Volcano plot showing PMA-induced DEGs in THP-1 cells. Vertical lines indicate − 1 or 1 of log_2_ fold change and the horizontal line indicates the *P*-value cut-off (*P* = 0.05). The dots indicate *UHRF1*, *DNMT1*, *DNMT3A*, *DNMT3B* and *TET1*. (**d**) (Left) qRT-PCR analysis to determine the mRNA level of *UHRF1* after PMA treatment in THP-1 cells. Data are shown as mean ± SEM (n = 3). The *P*-value was calculated by paired two-tailed t-test. ***P < 0.001. (Right) Western blot analysis to evaluate UHRF1 protein level in THP-1 after PMA treatment. (**e**) (Left) qRT-PCR analysis to determine the mRNA level of *DNMT1* after PMA treatment in THP-1 cells. Data are shown as mean ± SEM (n = 3). The *P*-value was calculated by paired two-tailed t-test. *P < 0.05. (Right) Western blot analysis to evaluate DNMT1 protein level in THP-1 after PMA treatment. (**f**) (Left) qRT-PCR analysis to determine the mRNA level of *DNMT3A* after PMA treatment in THP-1 cells. Data are shown as mean ± SEM (n = 3). The *P*-value was calculated by paired two-tailed t-test. n.s., not significant. (Right) Western blot analysis to evaluate DNMT3A protein level in THP-1 after PMA treatment. *non-specific band. (**g**) (Left) qRT-PCR analysis to determine the mRNA level of *DNMT3B* after PMA treatment in THP-1 cells. Data are shown as mean ± SEM (n = 3). The *P*-value was calculated by paired two-tailed t-test. **P < 0.01. (Right) Western blot analysis to evaluate DNMT3B protein level in THP-1 after PMA treatment.

THP-1 cell line is derived from the acute monocytic leukemia (AMoL) patient and phorbol-12-myristate-13-acetate (PMA) treatment to these cells induces differentiation into macrophages^2^. First, we adopted next generation sequencing (NGS) approach to analyze transcriptome changes during THP-1 differentiation. To this end, we performed RNA sequencing (RNA-seq) with THP-1 cells before and after PMA treatment (Fig. 1a). As a result, 6,369 genes were identified as differentially expressed genes (DEGs) after PMA treatment (Fig. 1b). Interestingly, *UHRF1*, *DNMT1* and *DNMT3B* were included in the 2,888 downregulated genes and *DNMT3A* was upregulated after PMA treatment (Fig. 1c). To validate these RNA-seq results, we analyzed mRNA and protein levels of these genes after differentiation. Consistently, *UHRF1*, *DNMT1* and *DNMT3B* showed downregulation in both mRNA and protein levels and *DNMT3A* showed slight increase in protein level (Fig. 1d–g). These results suggest that genes related to DNA methylation are regulated when differentiation occurs in THP-1 cells.

In addition, we found that mRNA levels of *CD14* and *CD11b*, which are well-known differentiation markers of macrophages^23^, increased after PMA treatment indicating that PMA treatment successfully induced differentiation into macrophages (Supplementary Fig. 1a). Also, the mRNA levels of *RGS1* and *MMP9* markedly increased after differentiation (Supplementary Fig. 1b). *RGS1* controls the GTPase activity of G-protein alpha subunits^24,25^, and *MMP9* is secreted by macrophages and required for inflammatory macrophage migration^26,27^. Conversely, the mRNA levels of *SPIB* and *SYTL1* decreased remarkably (Supplementary Fig. 1c). *SPIB* is related to the differentiation of dendritic cells rather than macrophages^28^ and *SYTL1* is known to promote leukemogenesis^29^. We also performed Gene Set Enrichment Analysis (GSEA) with RNA-seq data. The GSEA results showed that the gene sets related to macrophage differentiation, macrophage activation, interferon-γ (IFN-γ) response were enriched in the PMA-treated group (Supplementary Fig. 1d). IFN-γ is known to prime macrophages by downregulating miR-3473b expression to suppress phosphatase and tensin homolog (PTEN), which is required for the full activation of macrophages^30^. These results also validate our RNA-seq data. Together, these data suggest that PMA treatment induces macrophage-differentiation in THP-1 cells and genes responsible for DNA methylation are dynamically regulated during differentiation.

### Knockdown of UHRF1 or DNMT1 results in enhanced sensitivity to PMA and induces pro-inflammatory cytokines in THP-1 cells

**Figure 2:**
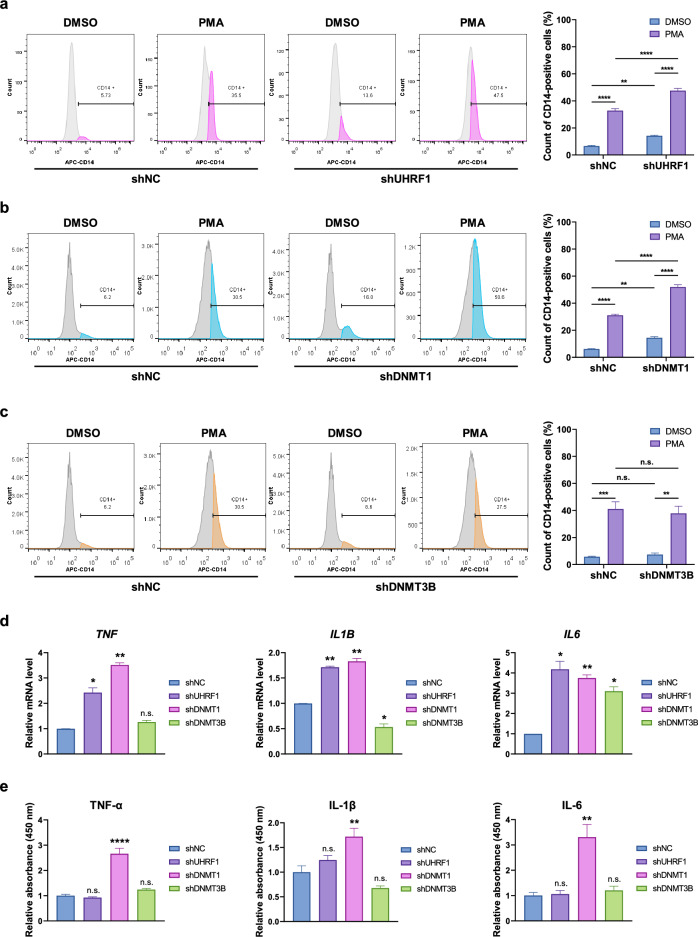
Depletion of UHRF1 or DNMT1 enhances sensitivity to PMA and upregulates cytokines related to M1 polarization in THP-1 cells. (**a**) FACS analysis to determine the counts of CD14-positive cells. Control and UHRF1 knockdown cells were treated with DMSO or PMA for 48 h. Data are shown as mean ± SEM (n = 3). The *P*-values were calculated by one-way ANOVA followed by Tukey’s multiple comparisons test. **P < 0.01 and ****P < 0.0001. (**b**) FACS analysis to determine the counts of CD14-positive cells. Control and DNMT1 knockdown cells were treated with DMSO or PMA for 48 h. Data are shown as mean ± SEM (n = 3). The *P*-values were calculated by one-way ANOVA followed by Tukey’s multiple comparisons test. **P < 0.01 and ****P < 0.0001. (**c**) FACS analysis to determine the counts of CD14-positive cells. Control and DNMT3B knockdown cells were treated with DMSO or PMA for 48 h. Data are shown as mean ± SEM (n = 3). The *P*-values were calculated by one-way ANOVA followed by Tukey’s multiple comparisons test. **P < 0.01, ***P < 0.001 and n.s., not significant. (**d**) qRT-PCR analysis to determine the mRNA level of *TNF*, *IL1B* and *IL6* in PMA-treated THP-1 cells. Cells were treated with PMA for 48 h and incubated for additional 48 h before harvest. Data are shown as mean ± SEM (n = 3). The *P*-values were calculated by one-way ANOVA followed by Dunnetts’s multiple comparisons test. *P < 0.05, **P < 0.01 and n.s., not significant. (**e**) Relative abundance of secreted TNF-α, IL-1β, IL-6 were analyzed by ELISA. Cells were treated with PMA for 48 h and incubated for additional 48 h before harvest. Relative absorbance at 450 nm were used for plotting. Data are shown as mean ± SEM (n = 3). The *P*-values were calculated by one-way ANOVA followed by Dunnetts’s multiple comparisons test. **P < 0.01, ****P < 0.0001 and n.s., not significant.

Since we found that expression levels of UHRF1, DNMT1, and DNMT3B decreased upon PMA treatment, we hypothesized that knockdown of these proteins could also affect PMA-induced differentiation in THP-1. We used short hairpin RNA (shRNA) to suppress expression of proteins and analyzed the CD14 surface marker by flow cytometry to evaluate the differentiation (Supplementary Fig. 2a). As expected, when control (shNC) cells were treated with PMA, the number of CD14-positive cells increased (Fig. 2a). Interestingly, the percentage of CD14-positive cells in shUHRF1 cells was greater than that in shNC cells after PMA treatment (Fig. 2a). Also, DNMT1-depleted cells showed the same results (Fig. 2b). However, knockdown of DNMT3B, which decreased after differentiation, did not affect PMA-induced differentiation (Fig. 2c). Intriguingly, knockdown of UHRF1 or DNMT1 induced CD14 expression regardless of PMA treatment (Fig. 2a,b). These data suggest that UHRF1 and DNMT1 inhibits the differentiation of THP-1 and depletion of these proteins enhances differentiation of THP-1 cells.

Since depletion of UHRF1 or DNMT1 enhanced PMA-induced differentiation in THP-1, we next tested the differentiation when both proteins are either inhibited or depleted. We adopted 5-aza-2′-deoxycytidine (DAC) for inhibition of DNMT1. DAC has been shown to induce differentiation of acute myeloid leukemia (AML) cell lines by inducing hypomethylation of *CEBPE* promoter CpG islands^31^. We treated DAC and PMA to shNC and shUHRF1 cells and analyzed differentiation. As expected, PMA and DAC treatment induced CD14 expression in both cells (Supplementary Fig. 2b). However, we were unable to find powerful synergistic effect of inhibiting both UHRF1 and DNMT1 in inducing differentiation. This is might due to the fact that UHRF1 is required for recruitment of DNMT1 to DNA, suggesting that they are on the same pathway^32^.

Next, we checked mRNA levels of pro-inflammatory cytokines such as TNF-α, IL-1β and IL-6, after differentiation since PMA induces differentiation into pro-inflammatory macrophages^33^. When PMA was treated to control and knockdown cells, mRNA levels of those three cytokines were increased in UHRF1- or DNMT1-knockdown cells (Fig. 2d). In addition, when lipopolysaccharide (LPS) was treated after differentiation, knockdown of DNMT1 remarkably increased LPS-induced *TNF* and *IL6* mRNA levels and restored downregulated *IL1B* mRNA level by LPS (Supplementary Fig. 2c). In addition, when we analyzed abundance of secreted cytokines in culture media, knockdown of DNMT1 remarkably increased secretion of three pro-inflammatory cytokines (Fig. 2e and Supplementary Fig. 2d). Taken together, these data suggest that UHRF1 and DNMT1 act as negative regulators of THP-1 differentiation and depletion of these negative regulators enhances the sensitivity of THP-1 to PMA and induces expression of pro-inflammatory cytokines.

In addition, to find out whether knockdown of UHRF1 or DNMT1 also enhances differentiation in other cell line, we used another blood cancer cell line U-937, which is obtained from histiocytic lymphoma. U-937 cells also differentiate into macrophages upon PMA treatment^34^. First, when we checked protein levels of UHRF1 and DNMTs after differentiation in U-937 cells, protein levels of UHRF1 and DNMT1 dramatically decreased (Supplementary Fig. 3a). Next, we depleted expression of UHRF1 and DNMT1 in U-937 cells and induced differentiation (Supplementary Fig. 3b). As a result, knockdown of either protein enhanced sensitivity to PMA (Supplementary Fig. 3c). In addition, knockdown of DNMT1 induced CD14 expression without PMA treatment. These results suggest that UHRF1 and DNMT1 also regulate differentiation in U-937 cells.

### UHRF1 and DNMT1 regulate macrophage differentiation- and activation-related gene expression in THP-1

**Figure 3:**
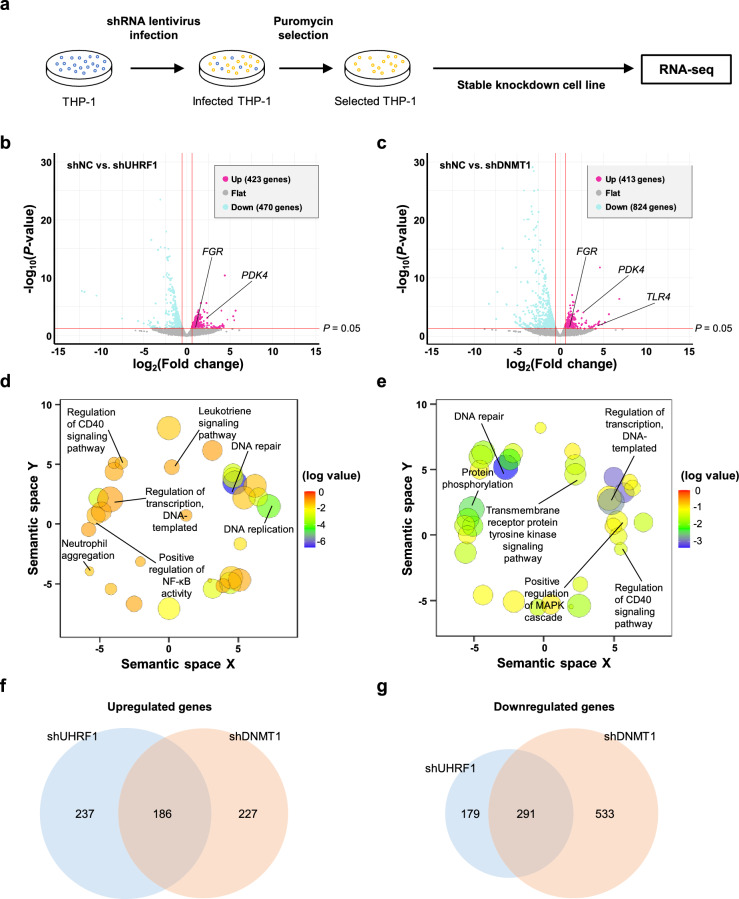
UHRF1 and DNMT1 regulates genes related to differentiation in THP-1 cells. (**a**) Schematic image of sample preparation process for RNA-seq. (**b**) Volcano plot representing DEGs induced by UHRF1 depletion in THP-1 cells. Vertical lines indicate the log_2_ fold change cut-off (|log_2_ fold change|> 0.585) and the horizontal line indicates the *P*-value cut-off (*P* = 0.05). (**c**) Volcano plot representing DEGs induced by DNMT1 depletion in THP-1 cells. The cut-off for DEG was same as in (**b**). (**d**) REVIGO plot representing GO analysis result of upregulated DEGs from (**b**). The color of the bubble indicates the log of the *P*-value for each GO term. The size of the circle indicates the frequency of the GO term in the underlying GO database. (**e**) REVIGO plot representing GO analysis results of upregulated DEGs from (**c**). (**f**) Venn diagram indicating overlapping upregulated DEGs in shUHRF1 and shDNMT1 cells. (**g**) Venn diagram indicating overlapping downregulated DEGs in shUHRF1 and shDNMT1 cells.

Since we found that knockdown of UHRF1 or DNMT1 increased sensitivity to PMA in THP-1 cells, we hypothesized that both UHRF1 and DNMT1 are deeply involved in regulation of THP-1 differentiation. To determine the functions of UHRF1 and DNMT1 in THP-1 more precisely, we performed RNA-seq with stable-knockdown THP-1 cells (Fig. 3a). When UHRF1 was depleted, we found that 470 genes were downregulated and 423 genes were upregulated (Fig. 3b). In case of DNMT1, we found that 824 genes were downregulated, and 413 genes were upregulated (Fig. 3c).

Next, we performed gene ontology (GO) analysis with upregulated genes (Fig. 3d, e). One study has shown that human monocytes isolated from the peripheral blood show DNA repair deficiency because they lack several DNA repair proteins, including XRCC1, ligase III, PARP-1, and DNA-PKcs, however, in macrophages derived from these monocytes, the levels of these proteins and DNA repair efficiency are recovered^35^. In addition, the CD40 signaling pathway is crucial for macrophage activation because it enhances the synthesis of pro-inflammatory cytokines and chemokines and upregulates the expression of major histocompatibility complex (MHC) class II and the co-stimulatory molecules CD80 and CD86^36^. Based on our data, the GO terms “DNA repair” and “regulation of the CD40 signaling pathway” were enriched in both shUHRF1 and shDNMT1 THP-1. NF-κB signaling is also important for several macrophage functions, including phagocytosis, bacterial killing, and antimicrobial peptide production^37^. In addition, macrophages can be activated directly by neutrophils, which in turn can be activated by cytokines and chemokines secreted by macrophages^38^. Furthermore, mitogen-activated protein kinases (MAPKs) are frequently activated in macrophages^39^. These result suggest that UHRF1 and DNMT1 regulate expression of genes related to macrophage differentiation and activation in THP-1 cells.

In DNMT3B-depleted THP-1, we found that 611 genes were upregulated, and 836 genes were downregulated (Supplementary Fig. 4a). However, the GO term analysis results of these 611 upregulated DEGs were found to be irrelevant to either differentiation or macrophage activation (Supplementary Fig. 4b). This result is consistent with our data that knockdown of DNMT3B depletion had no effect on the differentiation of THP-1 (Fig. 2c).

Next, we analyzed the upregulated DEGs both in shUHRF1 and shDNMT1 cells. A total of 186 genes showed increased expression when UHRF1 or DNMT1 was depleted (Fig. 3f and Supplementary Fig. 5a). GO analysis revealed that “DNA repair” was the top hit (Supplementary Fig. 5b). In addition, the GO term “activation of phospholipase C activity” was enriched after knockdown of UHRF1 or DNMT1. Phospholipase C signaling is important for mediating the inflammatory response and is involved in the activation of NF-κB, MAPK, and interferon regulatory factors^40^. When we analyzed overlapping downregulated DEGs both in shUHRF1 and shDNMT1 cells, 291 genes were identified (Fig. 3g and Supplementary Fig. 5c). GO analysis with these genes revealed that when UHRF1 or DNMT1 is depleted, biological processes related to immune response, response to interferons were downregulated (Supplementary Fig. 5d). The identified DEGs by RNA-seq were validated using qRT-PCR (Supplementary Fig. 5e,f). These findings suggest that UHRF1 and DNMT1 regulate the expression of genes related to macrophage differentiation and activation in THP-1 and depletion of UHRF1 or DNMT1 enhances expression of these genes.

### Knockdown of UHRF1 and DNMT1 also regulates gene expression after differentiation in THP-1 cells

**Figure 4:**
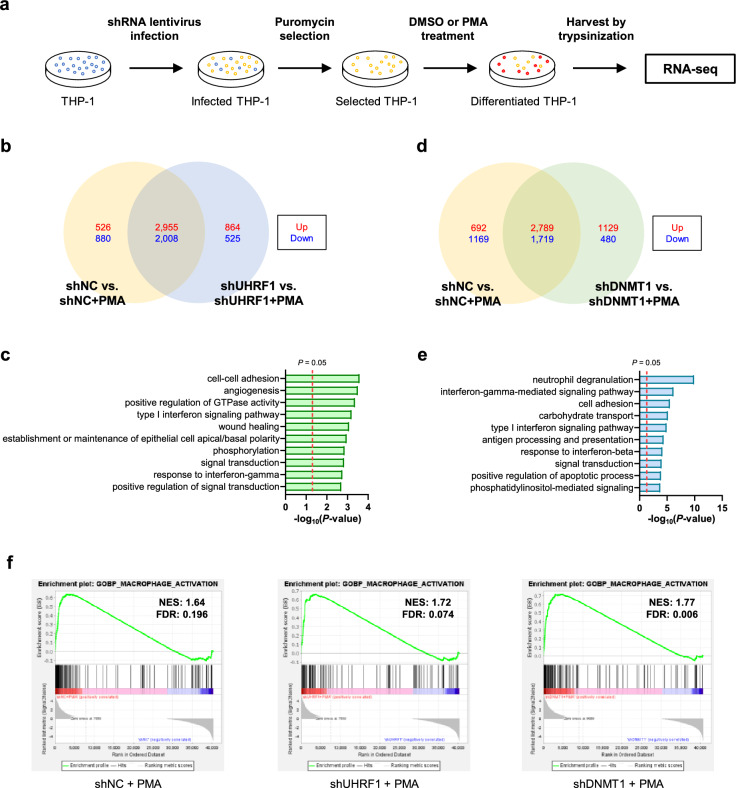
PMA treatment dynamically alters transcriptome in UHRF1- or DNMT1-depleted THP-1 cells. (**a**) Schematic image of sample preparation process for RNA-seq. Stable knockdown THP-1 cells were prepared and DMSO or PMA was added to selected THP-1 cells to induce differentiation. (**b**) Venn diagram showing counts of overlapping DEGs in RNA-seq results with indicated groups. Counts of upregulated or downregulated gene are indicated in red and blue, respectively. (**c**) Functional classification of 864 genes in (**b**) which were found out to be exclusively upregulated when PMA was treated to UHRF1 knockdown cells. The top 10 GO terms are shown as a bar graph. The dotted line indicates *P*-value of 0.05. (**d**) Venn diagram showing counts of overlapping DEGs in RNA-seq results with indicated groups. (**e**) Functional classification of 1129 genes in (**d**) which were found out to be exclusively upregulated when PMA was treated to DNMT1 knockdown cells. The top 10 GO terms are shown as a bar graph. The dotted line indicates *P*-value of 0.05. (**f**) Enrichment plot showing gene sets related to macrophage activation under each condition. NES: normalized enrichment score, FDR: false discovery rate.

To further investigate the roles of UHRF1 and DNMT1 in the differentiation process, we compared DEGs after inducing differentiation in control cells and UHRF1- or DNMT1-depleted cells (Fig. 4a). We identified 864 genes only upregulated in differentiated UHRF1-deficient cells (Fig. 4b). GO terms related to these 864 genes were similar to the GO analysis results of PMA-induced genes (Fig. 4c). One of the notable GO terms was “positive regulation of GTPase activity,” which is known to regulate the development and activation of macrophages^41,42^. In the case of DNMT1-deficient cells, 1129 genes were exclusively upregulated (Fig. 4d). The GO analysis result of these genes was also related to the immune response (Fig. 4e). The GO term “antigen processing and presentation” was one of the top ten hits. Macrophages are known to present antigens to initiate primary immune responses^43^. In addition, GSEA results showed that gene sets related to “macrophage activation” were enriched after the differentiation of shNC, shUHRF1, and shDNMT1 cells. However, the normalized enrichment score (NES) and false discovery rate (FDR) were more significant in UHRF1- or DNMT1-depleted cells (Fig. 4f). Taken together, these results indicate that knockdown of UHRF1 or DNMT1 also affects gene expression after PMA treatment in THP-1 cells and these changes are related to macrophage activation.

### Knockdown of DNMT1 abrogates global methylation status of differentiation-related genes in THP-1 cells

**Figure 5:**
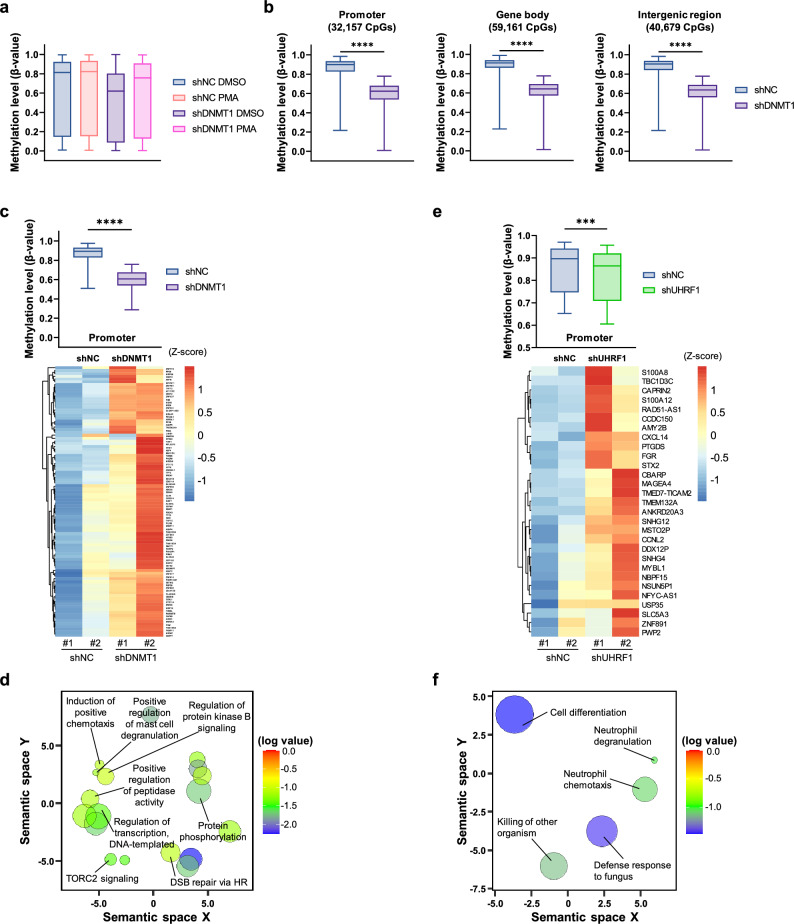
Knockdown of DNMT1 restrains global DNA methylation and regulates gene expression related to differentiation in THP-1 cells. (**a**) Box plot representing methylation level after BMIQ normalization of each sample. Methylation levels were analyzed by Illumina 850 K Methylation EPIC array. The minimum value, maximum value, and lower and upper quartiles were used for plotting. The line in the middle of each bar indicates the median value. For all cases, the *P*-values are less than 0.0001. The *P*-values were calculated by one-way ANOVA followed by Tukey’s multiple comparisons test. (**b**) Box plot showing the methylation level (β-value) of the hypomethylated CpG islands in DNMT1 knockdown THP-1 cells. The boxes extend from the 25th to the 75th percentiles and the line in the middle of the box indicates the median value. The *P*-values were calculated by unpaired two-tailed t-test. ****P < 0.0001. (**c**) Combined analysis of gene expression profiling and DNA methylation array in DNMT1 knockdown THP-1 cells. The genes which are upregulated and showed reduced promoter methylation level by DNMT1 depletion were used for plotting. The box plot represents 25th–75th percentiles with midlines indicating the median values. The heatmap was generated using the RPKM values for each gene. The *P*-values were calculated by paired two-tailed t-test. ****P < 0.0001. (**d**) Visualization of GO analysis results of the specified genes in (C). The color of the bubble indicates the log *P*-value for each GO term. The circle size indicates the frequency of the GO term in the underlying GO database. (**e**) Combined analysis of gene expression profiles and DNA methylation arrays in UHRF1-depleted THP-1 cells. The genes which are upregulated and showed reduced promoter methylation level by UHRF1 depletion were used for plotting. The box plot represents the 25th–75th percentiles, with midlines indicating the median values. The heat map was generated by RPKM values for each gene. The *P*-values were calculated by paired two-tailed t-test. ***P < 0.001. (**f**) Visualization of GO analysis results of the genes specified in (**e**). The color of the bubble indicates the log of the *P*-value for each GO term. The size of the circle indicates the frequency of the GO term in the underlying GO database.

Since we found that the expression levels of UHRF1, DNMT1, and DNMT3B decreased after differentiation in THP-1, we examined genome-wide DNA methylation pattern after depleting these proteins in THP-1 by Illumina 850 K EPIC Methylation Array. Only the DNMT1-depleted cells showed global downregulation of methylation level, whereas knockdown of UHRF1 or DNMT3B had no significant effect (Fig. 5a and Supplementary Fig. 6a–c). We expected that global methylation level to be decreased after differentiation since expression levels of DNMT1 and UHRF1 were downregulated. However, global methylation level did not change remarkably after differentiation. Interestingly, when we checked expression level of proteins related to demethylation of DNA with RNA-seq result (Fig. 1c), we were able to find out that expression level of *TET1*, which is critical in demethylation of DNA, was dramatically decreased after PMA treatment. This might be a compensatory action to maintain methylation status in THP-1 during differentiation.

When DNMT1 was depleted in THP-1 cells, 131,997 CpG islands were found out to be hypo-methylated. Among these, 32,157 were identified as promoter, 59,161 were identified as gene body, and 40,679 were identified as intergenic regions (Fig. 5b). In contrast, knockdown of UHRF1 or DNMT3B did not induce remarkable hypomethylation (Supplementary Fig. 6d). The classification of hypomethylated CpG islands is shown in Supplementary Fig. 6e.

It is widely accepted that promoter methylation is related to gene silencing^44^. Therefore, we hypothesized that the expression of genes with hypomethylated promoters after DNMT1 depletion would be activated. Notably, GO analysis result with hypomethylated promoters after DNMT1 knockdown showed that those genes were enriched in the terms “endocytosis,” “chemotaxis,” “inflammatory response,” “INF-γ-mediated signaling pathway,” “GTPase activity,” and “apoptosis,” which are closely related to activation and differentiation of macrophages (Supplementary Fig. 6f). This result suggests that inhibition of global DNA methylation in promoter regions by depletion of DNMT1 activates the expression of genes related to differentiation and macrophage activation.

Given that DNA methylation affects genomic integrity and transcriptional regulation, we compared the data from DNA methylation arrays with RNA-seq data. We analyzed CpG islands containing the promoters of upregulated genes when DNMT1 is depleted in the methylation array and found that the promoters of 96 genes were covered by the methylation array. The methylation levels of these promoters were remarkably lower than those in control cells (Fig. 5c). These data suggest that the expression of these genes is regulated by the methylation of their promoters by DNMT1. The GO terms related to these 96 genes were “induction of positive chemotaxis,” “regulation of transcription, DNA-templated,” “positive regulation of mast cell degranulation,” “protein phosphorylation,” and “TORC2 signaling” (Fig. 5d). TORC2 signaling is known to regulate proinflammatory macrophage polarization by suppressing Akt signaling^45^.

In case of shUHRF1 cells, we were able to identify 29 genes in the same way (Fig. 5e). GO analysis showed that these genes were related to “neutrophil degranulation,” “neutrophil chemotaxis,” and “killing of other organisms” and the top GO term was “cell differentiation” (Fig. 5f). Taken together, these data suggest that DNMT1 maintains global DNA methylation status and regulates differentiation-related gene expression in conjunction with UHRF1 to control sensitivity to PMA treatment in THP-1.

### Knockdown of UHRF1 or DNMT1 also inhibits THP-1-derived solid tumor growth in vivo

**Figure 6:**
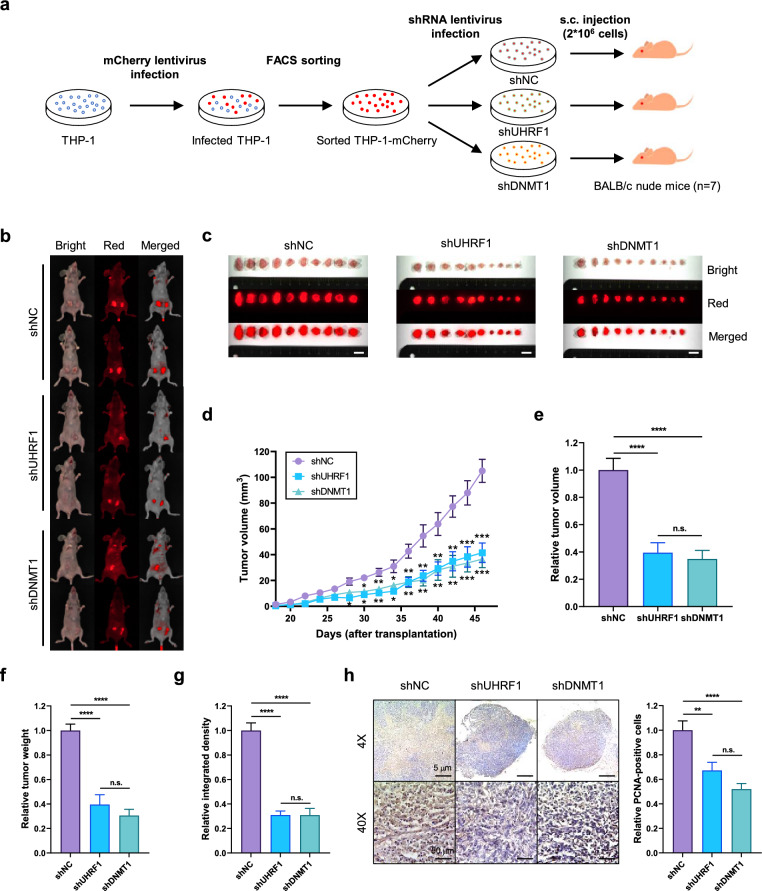
Depletion of UHRF1 or DNMT1 impedes THP-1-derived solid tumor growth in vivo. (**a**) Schematic image of strategy to generate THP-1 xenograft model. s.c, subcutaneous. (**b**) Representative fluorescent images of each group. The red fluorescent intensity was photographed by FOBI imager with anesthetized mice. (**c**) The red fluorescent intensity of tumors in each group was measured after mouse sacrifice. Scale bar = 1 cm. (**d**) The tumor volumes were determined every other day after xenotransplantation of different THP-1 cells. Data are shown as mean ± SEM (n = 10). The *P*-values were calculated by unpaired two-tailed t-test with control. *P < 0.05, **P < 0.01 and ***P < 0.001. (**e**) Tumor volumes were measured after mice sacrifice. All volumes were normalized to the mean of control group to obtain relative values. Data are shown as mean ± SEM (n = 10). The *P*-values were calculated by one-way ANOVA followed by Tukey’s multiple comparisons test. ****P < 0.0001 and n.s., not significant. (**f**) Weight of tumors was measured after mice sacrifice. All weights were normalized to the mean of control group to obtain relative values. Data are shown as mean ± SEM (n = 10). The *P*-values were calculated by one-way ANOVA followed by Tukey’s multiple comparisons test. ****P < 0.0001 and n.s., not significant. (**g**) Integrated density of red fluorescence was calculated by FOBI imager. All values were normalized to the mean of control group to obtain relative values. Data are shown as mean ± SEM (n = 10). The *P*-values were calculated by one-way ANOVA followed by Tukey’s multiple comparisons test. ****P < 0.0001 and n.s., not significant. (**h**) The differences in cell proliferation of shNC, shUHRF1 and shDNMT1 tumors were examined by immunohistochemistry of PCNA (left) and quantified data were shown on the right. Quantified data were calculated with 9 images in every group. Data are shown as mean ± SEM (n = 9). The *P*-values were calculated by one-way ANOVA followed by Tukey’s multiple comparisons test. **P < 0.01, ****P < 0.0001 and n.s., not significant. Scale bar = 5 μm.

To examine whether UHRF1 or DNMT1 also regulate proliferation of THP-1 cells in vivo, we designed THP-1 xenograft experiment (Fig. 6a and Supplementary Fig. 7a). Around 15–18 days after transplantation, tumors started to generate in each group (Fig. 6d). Mice were sacrificed when tumor volume in control group reached 100 mm^3^. Surprisingly, tumor growth was remarkably inhibited in shUHRF1 and shDNMT1 groups (Fig. 6b–d). When compared with control group, relative tumor volumes and weights of shUHRF1 and shDNMT1 groups decreased. (Fig. 6e,f). Also, intensity of red fluorescence was also weakened in both groups (Fig. 6g). In accordance with these data, PCNA-positive cells decreased in tumors derived from shUHRF1 and shDNMT1 cells indicating that the proportion of proliferating cells are smaller in those groups (Fig. 6h). Xenotransplantation of three different THP-1 cells had no significant effect on mouse body weight or major organ histology (Supplementary Fig. 7b,c). These results suggest that knockdown of UHRF1 or DNMT1 inhibits in vivo tumor growth derived from THP-1 cells.

### UHRF1 and DNMT1 are overexpressed in AML and related to poor prognosis of patients

**Figure 7:**
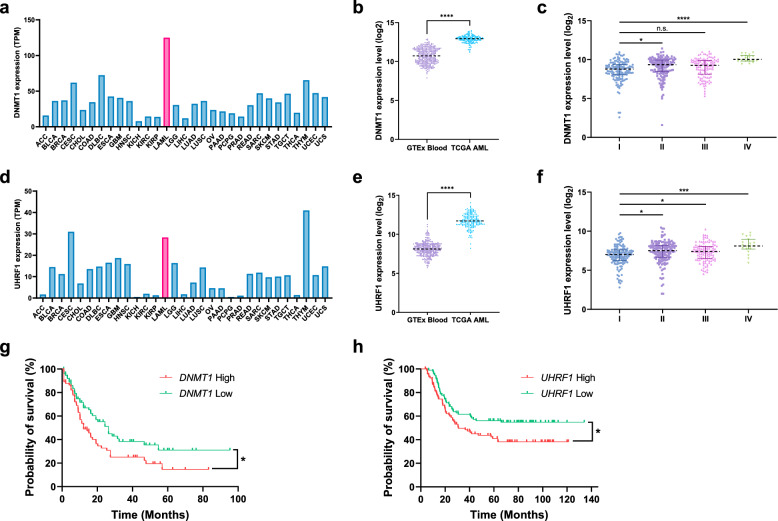
UHRF1 and DNMTs are overexpressed in AML and UHRF1 and DNMT1 are related to poor prognosis. (**a**) Bar graph showing the relative expression of *DNMT1* in 31 different cancer types by Gene Expression Profiling Interactive Analysis (GEPIA2). The heights of the bars represent the median expression for certain tumor types. (**b**) Scatter dot plot comparing the relative expression level of *DNMT1* in the GTEx blood (n = 337) and TCGA AML datasets (n = 173). This analysis was performed using the UCSC Xena browser. The y-axis shows the log_2_ value of the normalized RSEM count. The *P*-value was determined using a two-tailed t-test. ****P < 0.0001. (**c**) Analysis of *DNMT1* expression patterns in blood cancer stages (I [n = 139], II [n = 155], III [n = 113], and IV [n = 19]) in the Gene Expression database of Normal and Tumor tissues (GENT2). The y-axis shows the log_2_ value of relative mRNA expression levels. The *P*-values were determined using one-way ANOVA followed by Dunnett’s multiple comparisons test. *P < 0.05, ****P < 0.0001 and n.s., not significant. (**d**) Bar graph showing the relative expression of *DNMT1* in 31 different cancer types by GEPIA2. (**e**) Scatter dot plot comparing the relative expression level of *UHRF1* in the GTEx blood (n = 337) and TCGA AML datasets (n = 173). The *P*-value was determined using a two-tailed t-test. ****P < 0.0001. (**f**) Analysis of *UHRF1* expression patterns in blood cancer stages (I [n = 139], II [n = 155], III [n = 113], and IV [n = 19]) in the GENT2. The *P*-values were determined using one-way ANOVA followed by Dunnett’s multiple comparisons test. *P < 0.05 and ***P < 0.001. (**g**) Survival analysis of AML patients by OncoLnc. A total of 150 patients were divided into two groups (*DNMT1* high [n = 75] and *DNMT1* low [n = 75]) based on their expression level. The *P*-value was calculated by log-rank (Mantel–Cox) test. *P < 0.05. (**h**) Survival analysis of AML patients by UCSC Xena. A total of 187 patients were divided into two groups (*UHRF1* high [n = 93] and *UHRF1* low [n = 94]) based on their expression level. The *P*-value was calculated by log-rank (Mantel–Cox) test. *P < 0.05.

Aberrant DNA methylation is hallmark of many cancers^46,47^. Also, UHRF1 is frequently overexpressed in various cancers and exhibits oncogenic activity^48,49^. To further investigate whether expression of DNMT1 or UHRF1 is related to prognosis of AML patients, we examined expression pattern of these proteins in AML. First, we assessed the DNMT1 expression in 31 different cancers and found that expression in AML ranked the highest out of 31 cancer types (Fig. 7a). In addition, The Cancer Genome Atlas (TCGA) database analysis revealed that DNMT1 expression was highly upregulated in AML patients (Fig. 7b). Also, expression level of DNMT1 was positively correlated with blood cancer stage (Fig. 7c). Consistently, UHRF1 showed higher expression level in AML patients and higher stage of blood cancer (Fig. 7d–f). In addition, survival rates of AML patients were lower in high-expression groups of DNMT1 or UHRF1 (Fig. 7g,h). In case of DNMT3A and DNMT3B, they were found out to be overexpressed in AML patients but their expression levels were not positively correlated with blood cancer stage nor poor prognosis of AML patients (Supplementary Fig. 8a–h). These results suggest that DNMT1 and UHRF1 are frequently overexpressed in AML patients and might have possible role in deteriorating the disease and poor prognosis of AML patients.

Taken together, our data revealed that UHRF1 and DNMT1 play a crucial role in regulating differentiation of THP-1 cells by controlling genome-wide methylation status and transcription. The fact that depleting UHRF1 or DNMT1 enhances differentiation of THP-1 cells and inhibiting tumor growth in xenograft mice strongly suggest that targeting UHRF1 or DNMT1 might be a novel therapeutic strategy to treat MLL-AF9 AML by inhibiting cellular proliferation and inducing differentiation.

## Discussion

In this study, we showed that UHRF1 and DNMT1 regulate PMA-induced differentiation of THP-1 cells. We found that the expression of UHRF1 and DNMT1 decreased during THP-1 differentiation, and we validated our results using different methods, including RNA-seq. In this transcriptome analysis, we identified the changes in gene expression patterns when UHRF1 or DNMT1 was depleted in THP-1 cells. Specifically, we showed that depletion of UHRF1 or DNMT1 promotes THP-1 differentiation. Our analysis revealed dynamic transcriptome changes during the PMA-induced differentiation of THP-1 cells. In addition, using the DNA methylation array, we analyzed global DNA methylation patterns affected by the depletion of UHRF1 or DNMT1. All of these results suggest that UHRF1 and DNMT1 act as negative regulators of THP-1 differentiation by regulating DNA methylation and the expression of genes related to the differentiation and activation of macrophages.

The E3 ubiquitin ligase UHRF1 regulates epigenetic modifications and can recognize modifications in both DNA and histones^10^. By recognizing the presence of hemi-methylated DNA, UHRF1 maintains genomic DNA methylation levels by recruiting DNMT1 to DNA replication sites^50^. Consequently, depleting UHRF1 or DNMT1 inside the cell results in global DNA hypo-methylation and affects the expression patterns of their target genes. However, depletion of UHRF1 in THP-1 cells did not induce global hypo-methylation when we performed two independent arrays (Supplementary Fig. 6a,b). This result indicates that DNMT1 is predominant regulator of DNA methylation of genes related to differentiation in THP-1 cells.

In this study, we identified new target genes of UHRF1 and DNMT1 in THP-1 cells, which are highly related to the differentiation of THP-1 cells. The combined analysis of gene expression profiling and DNA methylation array results suggested that the differentiation of THP-1 cells upon PMA treatment was markedly promoted by suppressing the expression of UHRF1 or DNMT1 in THP-1 cells and that changes in differentiation- and macrophage activation-related gene expression caused by aberrant DNA methylation were responsible for these results.

Dysregulation of DNA methylation by genetic alterations in DNMTs or TETs disrupts normal hematopoiesis and eventually results in hematological malignancies, including AML^51^. Aberrant DNA methylation, a hallmark of AML, is an important epigenetic marker used for early diagnosis, prognostic prediction, and therapeutic decision-making^51^. Indeed, deletion of *DNMT1* prevents the development of MLL-AF9 leukemia in vivo, suggesting that MLL fusion proteins require maintenance methylation but not de novo methylation for leukemia induction^16^. DNA methylation analysis of THP-1 cells revealed that DNMT1 is crucial for THP-1 cells to maintain their leukemic properties, and knockdown of DNMT1 resulted in enhanced sensitivity to PMA treatment. These results are consistent with the fact that abnormal DNA methylation is a one of the major characteristics of AML.

In our previous study of aurora kinase A (AURKA) in THP-1 cells, we discovered that the expression of AURKA decreased during differentiation of THP-1 cells, and that pharmacological inhibition of AURKA activity resulted in enhanced differentiation by activating the KDM6B pathway^52^. In addition, we showed that AURKA knockdown induced the expression of CD14 without PMA treatment^52^. Intriguingly, we also found that knockdown of UHRF1 or DNMT1 increased the proportion of CD14-positive THP-1 cells, regardless of PMA treatment (Fig. 2a,b). This suggests that UHRF1 and DNMT1, similar AURKA, may play key roles in inhibiting THP-1 cell differentiation.

Surprisingly, co-regulation of UHRF1 and DNMT1 did not show synergistic effect on enhancing sensitivity to PMA in THP-1 cells. This unexpected outcome is probably due to the fact that DNMT1 and UHRF1 are on the same pathway in regulating DNA methylation or the excessive cellular toxicity from two different drugs, DAC and PMA, used in our experiment. Further studies are needed to discover possible synergistic partners for both DNMT1 or UHRF1 in regulating differentiation of THP-1 cells.

Knockdown of DNMT1 induced global hypomethylation in THP-1 cells, however, knockdown of UHRF1 did not (Fig. 5a and Supplementary Fig. S6a). To validate this result, we performed DNA methylation array once more with shUHRF1 cells and found out that global hypomethylation was not induced again (Supplementary Fig. S6b). Also, knockdown of DNMT3B did not affect global DNA methylation level (Supplementary Fig. S6c). Studies with mouse embryonic stem cells (mESCs) and colorectal cancer cells showed that knockdown of UHRF1 induced global DNA hypomethylation^32,53,54^. These results suggest the possibility that DNMT1 is predominant DNA methylation regulator in THP-1 cells which has more important roles in regulating global DNA methylation pattern than UHRF1 and DNMT3B.

Importantly, depletion of UHRF1 or DNMT1 significantly inhibited THP-1-derived solid tumor formation in vivo xenograft mouse, which strongly suggests the existence of a previously unidentified mechanisms of DNA methylome variation-associated leukemic cell differentiation.

Currently, inhibitors targeting epigenetic modulations such as isocitrate dehydrogenase (IDH), fms-like kinase 3 (FLT3), lysine-specific histone demethylase 1A (LSD1), DOT1-like protein (DOT1L) and bromodomain and extraterminal (BET) proteins are under investigation to treat AML patients^22,55^. However, reducing cytotoxicity of differentiation therapy agents to treat AML is a major task for developing new therapeutic strategies.

In conclusion, our study suggests that PMA-induced differentiation of THP-1 cells is promoted by the depletion of UHRF1 or DNMT1 which also reduced in vivo tumor growth. Therefore, inhibiting UHRF1 or DNMT1 expression may be a new therapeutic strategy. In addition, we suggest inhibiting DNMT1 and UHRF1 as promising therapeutic strategy for increasing the efficiency of leukemic cell differentiation to treat AML with differentiation therapy agents.

## Materials and methods

### Cell culture and differentiation induction

THP-1 and U-937 cells were grown in RPMI-1640 (Gibco, Waltham, MA, USA) containing 10% heat-inactivated fetal bovine serum (Gibco) and 0.05% penicillin–streptomycin (Welgene, Gyeongsan, Korea) at 37 °C in 5% CO_2_ atmosphere. HEK293T cells were grown in Dulbecco’s modified Eagle’s medium (DMEM) (Gibco) under the same conditions. Cells were purchased from Korean Cell Line Bank (Seoul, Korea). To induce the differentiation of THP-1 and U-937 cells, 100 ng/ml phorbol-12-myristate-13-acetate (PMA) or 1 µg/ml 5-aza-2′-deoxycytidine (DAC) (Sigma-Aldrich, St. Louis, MO, USA) was added and incubated for 48 h. Then cells were harvested by trypsinization and used for further experiments. DMSO (Duchefa, Haarlem, Netherlands) was used as the negative control for drug treatment.

### Stable knockdown cell lines

Target sequences of the short hairpin RNA (shRNA) for UHRF1, DNMT1 and DNMT3B are listed in Supplementary Table 1. The oligonucleotide containing target sequence was inserted into the AgeI/EcoRI site of the pLKO.1-TRC vector (Addgene, Watertown, MA, USA, #8453) according to standard protocols. To produce virus particles, we co-transfected the pLKO.1-TRC vector containing a specific shRNA sequence with the VSV-G envelope expressing plasmid pMD2.G (Addgene, #12259) and lentiviral packaging plasmid psPAX2 (Addgene, #12260) into HEK293T cells. Supernatants, including virus particles, were harvested 2 and 3 days after transfection and were used for viral infection with 8 µg/ml hexadimethrine bromide (Sigma-Aldrich). Twenty-four hours after infection, 1 µg/ml puromycin (Sigma-Aldrich) was used to positively select for infected cells.

### Quantitative reverse transcription-PCR (qRT-PCR)

Total RNA was extracted using the Tri-RNA reagent (Favorgen, Pingtung, Taiwan) according to the manufacturer’s protocol. To synthesize complementary DNA (cDNA), 1 μg of RNA was used. First, the RNA was incubated with oligo dT primers (Invitrogen, Waltham, MA, USA) at 70 °C for 5 min. Next, M-MLV reverse transcriptase (Enzynomics, Daejeon, Korea) and dNTPs were added. After incubation at 42 °C for 60 min, the enzyme was inactivated by incubating the samples at 95 °C for 30 s. qRT-PCR was performed using the SYBR® Green Supermix Kit (Takara, Kusatsu, Japan). Then, 39 cycles of amplification were performed. Each cycle included denaturation at 94 °C, annealing at temperatures specific for each primer set, and extension at 72 °C. Each experiment was performed in triplicate, and the mean threshold cycle (Ct) and standard error were calculated using the Ct value for each sample. The normalized mean Ct value (ΔCt) was calculated by subtracting the mean Ct value for glyceraldehyde 3-phosphate dehydrogenase (GAPDH) from that of the target gene. The ΔΔCt value was calculated as the difference between the control ΔCt and the ΔCt values for each sample. Finally, the n-fold change in mRNA levels was calculated as 2^−ΔΔCt^. Primer sets used for qRT-PCR are listed in Supplementary Table 2.

### Fluorescence activated cell sorting (FACS) analysis

FACS was used to measure the differentiation of THP-1, U-937 cells. To measure cell differentiation, cells were harvested after PMA treatment for 48 h. The cells were resuspended in cold phosphate-buffered saline (PBS) containing 1 mM EDTA, 1% bovine serum albumin (CellNest, Hanam, Korea), and 10 mM sodium azide for 1 h. The cells were then stained with APC-CD14 (#17-0149-42, Invitrogen) for 30 min and subjected to FACS analysis using the BD Accuri C6 Plus Flow Cytometer (BD Biosciences, Franklin Lakes, NJ, USA).

### Western blotting and antibodies

Harvested cells were lysed with the RIPA buffer (50 mM Tris–HCl [pH 8.0], 150 mM NaCl, 0.1% SDS, 0.5% SDC, 1% NP-40, 1× protease inhibitor cocktail, 1 mM EDTA [pH 8.0]). After incubating the cells in RIPA buffer for 1 h with gentle agitation at 4 °C, cell lysates were purified by centrifugation at 14,000 rpm for 15 min at 4 °C. Thirty micrograms of each lysate were subjected to sodium dodecyl sulfate–polyacrylamide gel electrophoresis (SDS-PAGE). After SDS-PAGE, western blotting was performed using the following antibodies. Antibodies against UHRF1 (sc-373750, 1:1000), β-actin (sc-47778, 1:1000) (Santa Cruz Biotechnology, Dallas, TX, USA), GAPDH (CSB-PA00025A0Rb, 1:5000) (Cusabio Technology, Houston, TX, USA), DNMT1 (#5032, 1:5000), DNMT3B (#57868, 1:5000, Cell Signaling Technology, Danvers, MA, USA) and DNMT3A (PA3-16557, 1:5000, Invitrogen) were used. β-actin (ACTIN) or glyceraldehyde-3-phosphate dehydrogenase (GAPDH) was used as loading control.

### RNA sequencing (RNA-seq)

For RNA-seq, 10 duplicate samples (THP-1 wild-type, shNC, shUHRF1, shDNMT1, and shDNMT3B cells before and after differentiation by PMA treatment) were prepared. Whole RNA-seq processes were performed by Thergen Bio Inc. (Seongnam, Korea). Libraries were prepared for 150 bp paired-end sequencing using the TruSeq Stranded mRNA Sample Prep Kit (Illumina, San Diego, CA, USA). mRNA molecules were purified and fragmented from 0.1 to 1 μg of total RNA using oligo (dT) magnetic beads. Fragmented mRNAs were synthesized as single-stranded cDNAs through random hexamer priming. Double-stranded cDNA was prepared by using this cDNA as a template for second strand synthesis. After a sequential process of end repair, A-tailing, and adapter ligation, cDNA libraries were amplified using PCR. The quality of these cDNA libraries was evaluated using the Agilent 2100 BioAnalyzer (Agilent, Santa Clara, CA, USA). They were quantified using the KAPA Library Quantification Kit (Kapa Biosystems, Wilmington, MA, USA) according to the manufacturer’s library quantification protocol. Following cluster amplification of denatured templates, sequencing was performed using paired-end (2 × 150 bp) reads using Illumina NovaSeq 6000 (Illumina). Low-quality reads were filtered based on the following criteria: reads containing more than 10% of skipped bases (marked as ‘N’s), reads containing more than 40% of bases with quality scores of less than 20, and reads with average quality scores for each read of less than 20. The filtering process was performed using in-house scripts. Filtered reads were mapped to the reference genome (hg38) related to the species using the aligner STAR v.2.4.0b. Gene expression levels were measured using Cufflinks v2.1.1 and the gene annotation database of the species. For differentially expressed gene (DEG) analysis, gene-level count data were generated using HTSeq-count v0.6.1p1. Based on the calculated read count data, DEGs were identified using the R package ‘TCC’. The normalization factors were calculated using the iterative DEGES/edgeR method. The q-value was calculated based on the *P*-value using the p.adjust function of the R package, with default parameter settings. The DEGs were identified based on a q-value threshold of less than 0.05 for correcting errors caused by multiple testing. Based on DEG analysis results, gene ontology (GO) analysis was performed using DAVID 2021^56,57^ or ShinyGO v0.75^58^. To visualize the GO analysis results, REVIGO^59^ was used. All plots and visualizations were generated using R 4.1.2 (https://www.r-project.org/). Gene set enrichment analysis (GSEA) was performed using GSEA v4.1.0 software^60,61^.

### Enzyme-linked immunosorbent assay (ELISA)

For ELISA, ELISA MAX™ Deluxe Set Human TNF-α (#430204), IL-1β (#437004) and IL-6 (#430504, BioLegend, San Diego, CA, USA) were used. Stable knockdown THP-1 cells (4 × 10^6^) were treated with PMA. After 48 h, media were removed and replaced with fresh media with or without 1 µg/ml of lipopolysaccharide (L2630, Sigma-Aldrich) and incubated for additional 48 h. Culture media were transferred to polypropylene tube and used for ELISA at 1:5 dilution ratio. ELISA was performed according to the manufacturer’s protocol and optical density at 450 nm was used for comparing relative abundance of the secreted cytokines.

### Public database analysis

The relative expression data of UHRF1, DNMT1, DNMT3A, and DNMT3B in normal and cancerous tissues or in specific cancer stages were analyzed using various platforms, including UCSC Xena^62^, Gene Expression database of Normal and Tumor tissues 2 (GENT2)^63^, Gene Expression Profiling Interactive Analysis (GEPIA2)^64^, and The University of Alabama at Birmingham Cancer Data Analysis Portal (UALCAN)^65^. Survival analyses were performed using the same online tools and OncoLnc^66^.

### Illumina 850 K methylation EPIC array

Genomic DNA (gDNA) was isolated from stable knockdown THP-1 cells before and after differentiation using the Wizard^®^ Genomic DNA Purification Kit (Promega, Madison, WI, USA). The quality of the DNA samples was evaluated using the NanoDrop^®^ ND-1000 UV–Vis spectrophotometer (Thermo Fisher Scientific, Waltham, MA, USA). Samples with intact gDNA that showed no smearing after agarose gel electrophoresis were selected for the experiment. Intact gDNA was quantified using Quant-iT™ PicoGreen™ (Invitrogen) diluted to a concentration of 50 ng/µl. The concentrations were adjusted based on these results. All prepared samples were bisulfite-converted using the Zymo EZ DNA Methylation Kit (Zymo Research, Irvine, CA, USA). For bisulfite conversion, 550 ng of input gDNA was used. The whole-genome amplification process required 200 ng of input bisulfite-converted gDNA, MA1, RPM, and MSM. This process created a sufficient quantity of DNA (1000X amplification) to be used on a single Infinium MethylationEPIC BeadChip Kit (Illumina). After amplification, the product was fragmented using a proprietary reagent (FMS), precipitated with 2-propanol (plus a precipitating reagent PM1), and resuspended in a formamide-containing hybridization buffer (RA1). The DNA samples were denatured at 95 °C for 20 min and then placed in a humidified container for a minimum of 16 h at 48 °C, allowing CpG loci to hybridize to the 50-mer capture probes. Following hybridization, the BeadChip/Te-Flow chamber assembly was placed on a temperature-controlled Tecan Flowthrough Chamber Rack (Tecan, Switzerland), and all subsequent washing, extension, and staining were performed by adding reagents to the chamber. For the allele-specific single-base extension assay, primers were extended with a polymerase and labeled nucleotide mix (TEM) and then stained with repeated application of a staining reagent (STM) and an anti-staining reagent (ATM). After completing the staining process, the slides were washed with a low-salt buffer (PB1), immediately coated with XC4, and then imaged using a two-color (532 nm/658 nm) confocal fluorescent scanner iScan System (Illumina). The image intensities were extracted using the iScan Control software (Illumina). Array data export processing and analysis were performed using Illumina GenomeStudio v2011.1 (Methylation Module v1.9.0) and R 3.3.3 (http://www.r-project.org). The multidimensional scaling (MDS) analysis was performed with Euclidean distance. The Illumina Infinium Methylation EPIC array covers over 850,000 CpG islands for DNA methylation. These CpG islands were categorized according to the UCSC RefGene group into promoters, gene bodies, and intergenic regions.

### Tumor xenograft establishment and tissue section preparation

All experiments were performed in accordance with the ARRIVE guidelines (https://arriveguidelines.org) and were carried out in accordance with the relevant guidelines and regulations. Four-week-old BALB/c nu/nu immunodeficient mice (weights ranged between 15 and 18 g) (Orient Bio, Seongnam, Korea) were randomly allocated into two groups (seven mice in each group). The control, UHRF1 or DNMT1 stably knockdown mCherry expressing THP-1 cell (2 × 10^6^) suspension in 0.1 ml 1:1 (v/v) mixture of Matrigel (Corning, Tewksbury, MA, USA) and PBS were subcutaneously injected into mice. Three weeks after xenotransplantation, the body weights and tumor sizes were measured every other day. The volume (mm^3^) of each tumor ((length × width^2^ × π)/6) was determined. Tumor-bearing mice were sacrificed by cervical dislocation after continuous monitoring of tumor growth for 3 weeks. The fluorescence density of the xenograft tumors was photographed and analyzed by FOBI Fluorescence In Vivo Imaging System (CELLGENTEK, Cheongju, Korea) after anesthetizing with ketamine/xylazine. Tumor, liver, kidney, spleen, lung and heart tissues were excised and fixed in 4% paraformaldehyde (Sigma-Aldrich) for 24 h, followed by dehydration in 30% sucrose in PBS until tissue sinks. Next, tissues were embedded in FSC22 Frozen Section Compound (Leica Microsystems, Richmond, IL, USA) and 10‐μm sections were sliced using a Leica CM1950 Cryostat (Leica Biosystems, Wetzlar, Germany). Tissue sections were mounted onto poly-L-lysine (Sigma-Aldrich) coated slides and stored at -80 °C for later staining. The animals were treated as described in the protocol and no blinding method was used for animal studies. There were no animal exclusion criteria.

### Immunohistochemistry

The tumor sections were fixed in acetone at -20 °C for 10 min and followed by the antigen retrieval by boiling in 0.01 M Sodium citrate buffer (pH 6.0) for 20 min, and permeabilization using 0.2% Triton X-100 for 15 min. To detect the cell proliferation in tumors, immunohistochemistry of proliferating cell nuclear antigen (PCNA) was applied. Permeabilized tissues were incubated with 3% hydrogen peroxidase for 10 min followed by 1 h of non-specific binding blocking by 2.5% normal goat serum (Agilent). The slides were incubated with primary proliferating nuclear antigen antibody (1:100) in antibody dilute solution (Agilent) at 4 °C overnight followed by rinsing with PBS-T and incubated with HRP conjugated anti-mouse IgG polyclonal antibody (Enzo Life Sciences, Farmingdale, NY, USA). Slides were washed with PBS-T, and color was developed using 3,3′-diaminobenzidine (DAB). The slides were counterstained with hematoxylin followed by cover-slipping with Mountant (Thermo Fisher Scientific), examined and photographed using Leica ICC50 HD camera (Leica Microsystems, Wetzlar, Germany). PCNA labeling was quantified using Image J software^67^.

### Hematoxylin and eosin staining

Frozen tissue sections were fixed in acetone at -20 °C for 3 min and 80% methanol at 4 °C for 4 min followed by hematoxylin staining for 2 min and washed with distilled water for 5 min. Slides were labelled with eosin for 30 s and dehydrated with gradient ethanol and cleared with xylene followed by cover-slipping with Mountant (Thermo Fisher Scientific).

### Statistical analysis

For two-group comparison, the unpaired, two-tailed Student’s t-test was used. For more than three-group comparisons, the one-way analysis of variance model (ANOVA) was performed followed by appropriate multiple comparisons method. The *P*-values for survival analyses were determined by the log-rank (Mantel–Cox) test and statistical analyses and visualization were performed using GraphPad Prism (GraphPad Software, San Diego, CA, USA). Data shown as bar graphs represent the mean and standard error of the mean (SEM) of mentioned replicates (at least three). The variance was similar between the groups that were being statistically compared. The *P*-values under 0.05 were considered to be statistically significant.

### Ethics declarations

This study was performed according to the Animal guidelines approved by the Chung-Ang University Institutional Animal Care and Use Committee (IRB# CAU2020-00,115).

### Supplementary Information

Supplementary Information.

## Data Availability

The data that support the findings of this study are openly available in NCBI Gene Expression Omnibus (GEO) with reference number GSE206742 (RNA-seq) or GSE206620 (Illumina Infinium Methylation EPIC array).
