# Supplementary material for: Downregulation of DNA methylation enhances differentiation of THP-1 cells and induces M1 polarization of differentiated macrophages
Source: Sci Rep. 2023 Aug 12;13:13132. doi: 10.1038/s41598-023-40362-8 (PMC10423279; doi:10.1038/s41598-023-40362-8)
Supplement: Supplementary file 1 — Supplementary Information. [file 41598_2023_40362_MOESM1_ESM.pdf]

SUPPLEMENTARY INFORMATION

for

Downregulation of DNA methylation enhances differentiation of THP-1 cells and induces M1 polarization of differentiated macrophages

Junyoung Park[1], Yongyang Luo[2], Jin Woo Park[1], Song Hyun Kim[1], Ye Joo Hong[1], Younghyun Lim[1], Young-Jin Seo[1], Jeehyeon Bae[2], and Sang Beom Seo[1,*]

[1] Department of Life Science, College of Natural Sciences, Chung-Ang University, Seoul 06974, Republic of Korea

[2] College of Pharmacy, Chung-Ang University, Seoul 06974, Republic of Korea

Supplementary Fig 1. PMA treatment induces dynamic transcriptome variations in THP-1 cells.

Supplementary Fig 2. Co-regulating UHRF1 and DNMT1 has little synergistic effect on differentiation and knockdown of UHRF1 or DNMT1 induces M1 polarization-related cytokines in THP-1 cells.

Supplementary Fig 3. Knockdown of UHRF1 or DNMT1 also enhances PMA-induced differentiation of U-937 cells.

Supplementary Fig 4. DNMT3B does not regulate genes related to differentiation in THP-1 cells unlike UHRF1 or DNMT1.

Supplementary Fig 5. Knockdown of UHRF1 or DNMT1 regulates gene expression in THP-1 cells.

Supplementary Fig 6. Knockdown of UHRF1, DNMT1 and DNMT3B each induces changes in DNA methylation pattern in THP-1 cells.

Supplementary Fig 7. Xenotransplantation of different THP-1 cell lines has no effect on body weight and major organ histology.

Supplementary Fig 8. Expression level of DNMT3A and DNMT3B are not related to poor prognosis of AML patients.

Supplementary Table 1. List of shRNA target sequences used in this study.

Supplementary Table 2. List of qRT-PCR primer sets used in this study.

Full images for western blots

Supplementary Fig 1

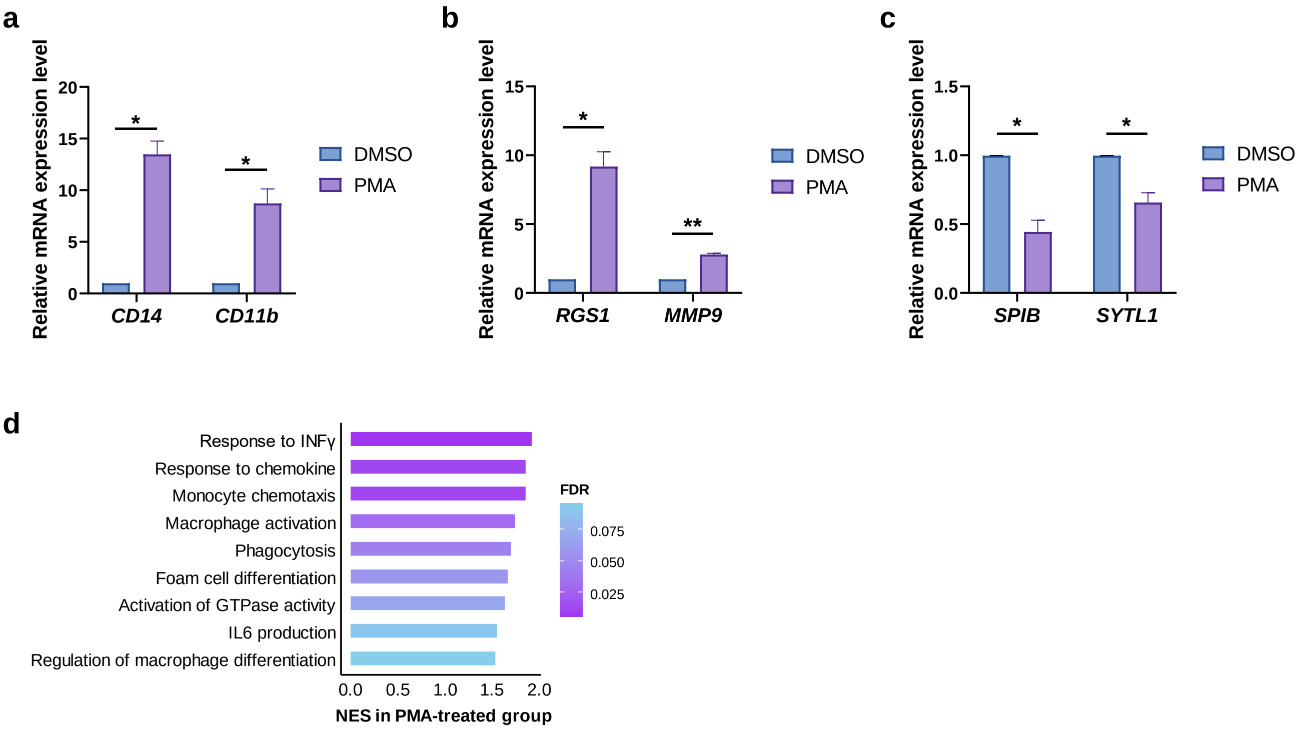

Supplementary Fig 1. PMA treatment induces dynamic transcriptome variations in THP-1 cells.

a. qRT-PCR analysis to determine the mRNA level of CD14 and CD11b, which are known as macrophage differentiation markers, after PMA treatment in THP-1 cells. Data are shown as mean ± SEM (n = 3). The *P*-value was calculated by paired two-tailed t-test. *$P < 0.05$.

b. qRT-PCR analysis to determine the mRNA level of RGS1 and MMP9, which are found out to be upregulated by PMA in RNA-seq, after PMA treatment in THP-1 cells. Data are shown as mean ± SEM (n = 3). The *P*-value was calculated by paired two-tailed t-test. *$P < 0.05$ and **$P < 0.01$.

c. qRT-PCR analysis to determine the mRNA level of SPIB and SYTL1, which are found out to be downregulated by PMA in RNA-seq, after PMA treatment in THP-1 cells. Data are shown as mean ± SEM (n = 3). The *P*-value was calculated by paired two-tailed t-test. *$P < 0.05$.

d. GSEA (Gene Set Enrichment Analysis) result is shown as a bar graph. Read count data from RNA-seq with DMSO- or PMA-treated THP-1 cells were used as input. Gene sets related to macrophage activation or differentiation were plotted. The x-axis indicates the normalized enrichment score (NES), and the color of the bar indicates the false discovery rate (FDR) value for each gene set.

Supplementary Fig 2

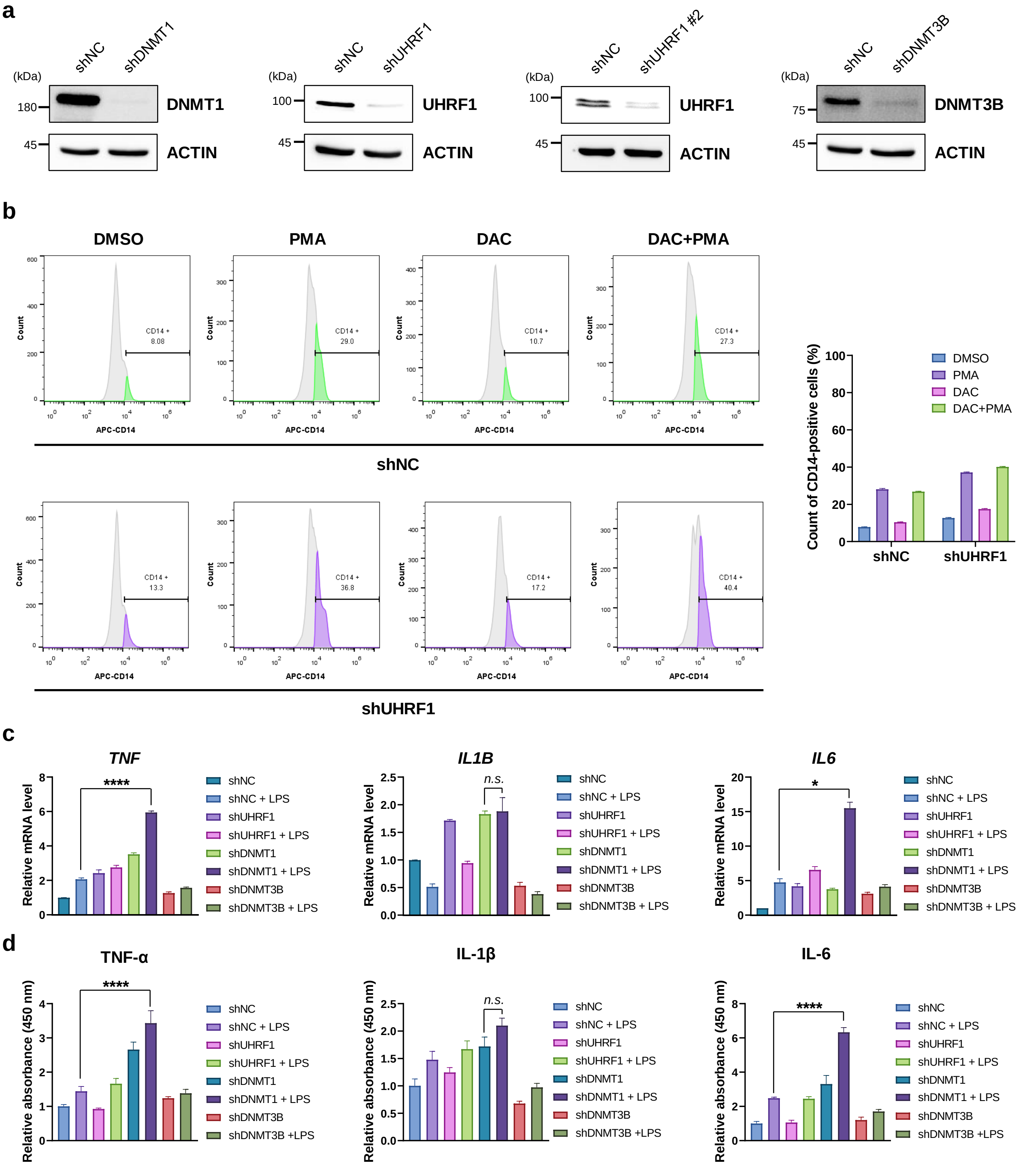

Supplementary Fig 2. Co-regulating UHRF1 and DNMT1 has little synergistic effect on differentiation and knockdown of UHRF1 or DNMT1 induces M1 polarization-related cytokines in THP-1 cells.

a. Western blot analysis to validate of the knockdown efficiency of target proteins in sample used for the following experiments.

Supplementary Fig 2

b. FACS analysis to determine the counts of CD14-positive cells. Control and UHRF1 knockdown cells were treated with DMSO or PMA or DAC (5-Aza-2'-deoxycytidine) for 48 h. Data are shown as mean ± SEM (n = 3).

c. qRT-PCR analysis to determine the mRNA level of TNF, IL1B and IL6 in PMA-treated THP-1 cells. Cells were treated with PMA for 48 h and incubated for additional 48 h with or without LPS (lipopolysaccharide) before harvest. Data are shown as mean ± SEM (n = 3). The *P*-values were calculated by one-way ANOVA followed by Tukey's multiple comparisons test. *$P < 0.05$, ****$P < 0.0001$ and n.s., not significant.

d. Relative abundance of secreted TNF-α, IL-1β, IL-6 were analyzed by ELISA. Cells were treated with PMA for 48 h and incubated for additional 48 h before harvest. Relative absorbance at 450 nm were used for plotting. Data are shown as mean ± SEM (n = 3). The *P*-values were calculated by one-way ANOVA followed by Tukey's multiple comparisons test. ****$P < 0.0001$ and n.s., not significant.

Supplementary Fig 3

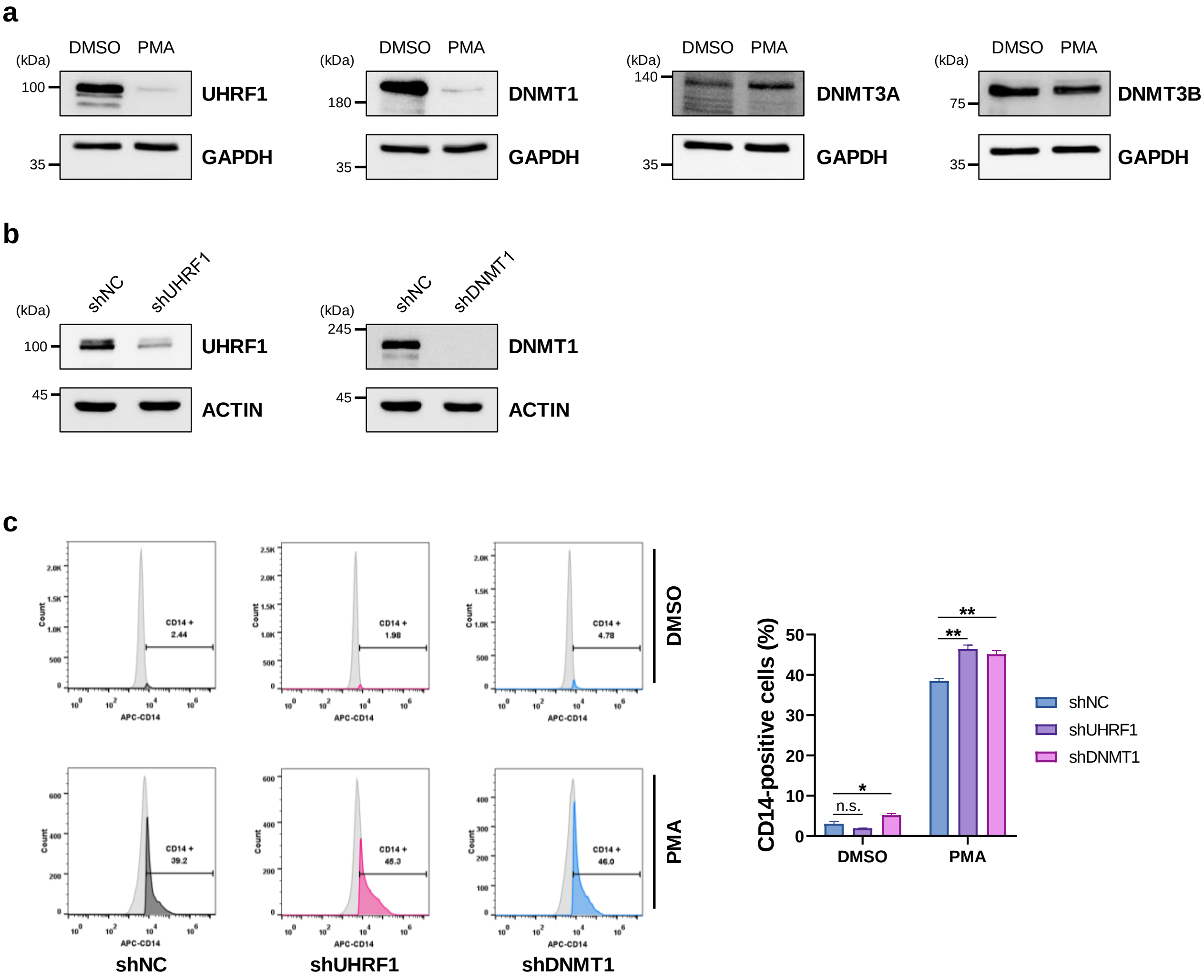

Supplementary Fig 3. Knockdown of UHRF1 or DNMT1 also enhances PMA-induced differentiation of U-937 cells.

a. Western blot analysis to examine protein levels of UHRF1 and DNMTs in differentiated U-937 cells.

b. Western blot analysis to validate of the knockdown efficiency of target proteins in sample used for the following experiments.

c. FACS analysis to determine the counts of CD14-positive cells. Control and UHRF1, DNMT1 knockdown cells were treated with DMSO or PMA for 48 h. Data are shown as mean ± SEM (n = 3). The *P*-values were calculated by one-way ANOVA followed by Tukey's multiple comparisons test. *$P < 0.05$, **$P < 0.01$ and n.s., not significant.

Supplementary Fig 4

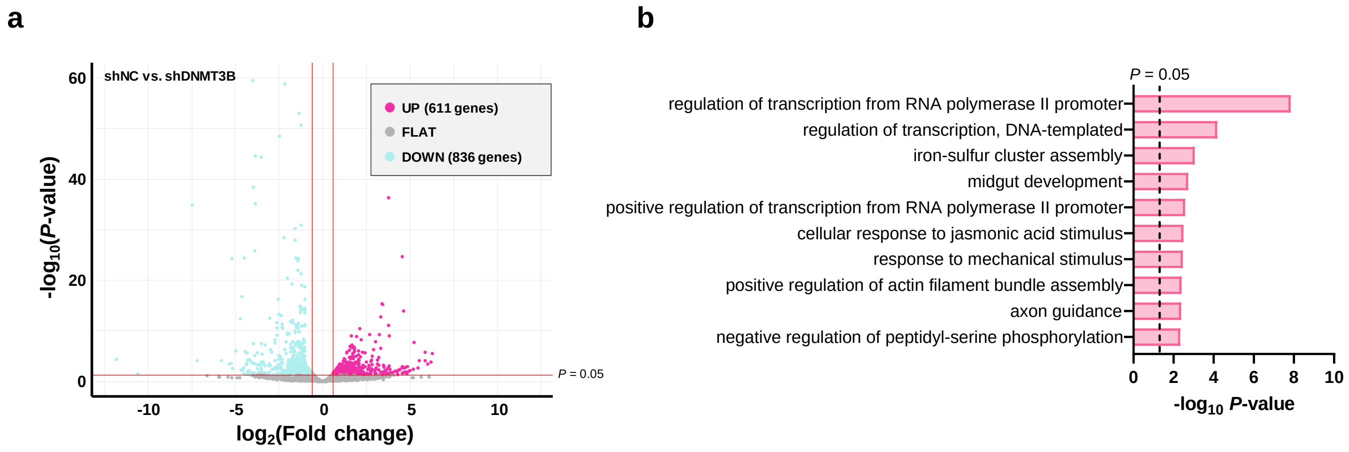

Supplementary Fig 4. DNMT3B does not regulate genes related to differentiation in THP-1 cells unlike UHRF1 or DNMT1.

a. Volcano plot representing DEGs induced by DNMT3B depletion in THP-1 cells. Vertical lines indicate the $\log_2$ fold change cut-off ($|\log_2$ fold change$| > 0.585$) and the horizontal line indicates the *P*-value cut-off ($P = 0.05$).

b. Bar graph showing GO analysis result of 611 upregulated DEGs induced by DNMT3B depletion. The dotted line indicates *P*-value of 0.05.

Supplementary Fig 5

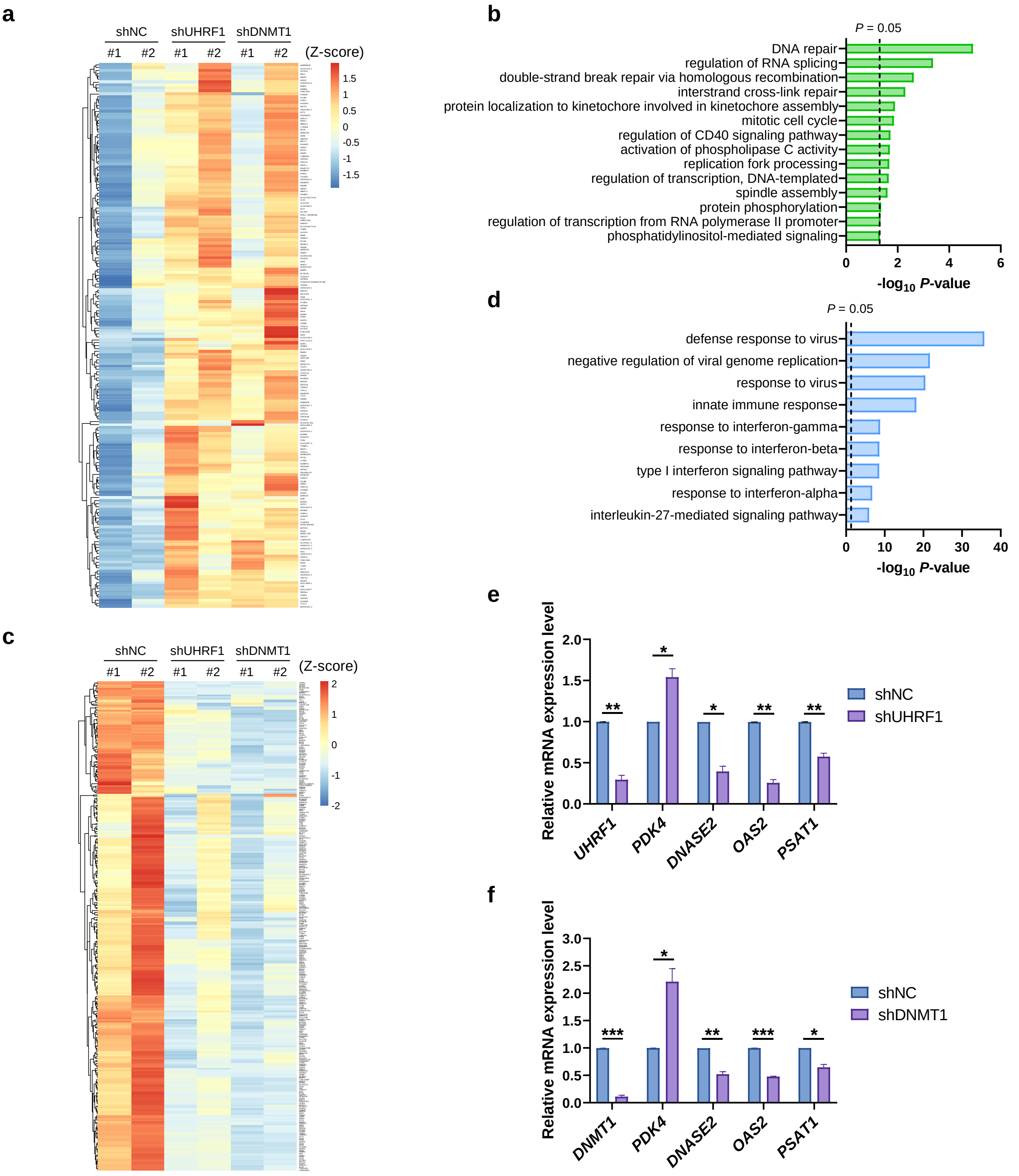

Supplementary Fig 5. Knockdown of UHRF1 or DNMT1 regulates gene expression in THP-1 cells.

a. Heatmap representing overlapping upregulated DEGs after UHRF1 or DNMT1 depletion in THP-1 cells.

b. Bar graph showing GO analysis result with genes in (A). The dotted line indicates *P*-value of 0.05.

Supplementary Fig 5

c. Heatmap representing overlapping downregulated DEGs after UHRF1 or DNMT1 depletion in THP-1 cells.

d. Bar graph showing GO analysis result with genes in (C). The dotted line indicates *P*-value of 0.05.

e. qRT-PCR analysis to validate the RNA-seq results in UHRF1-depleted THP-1 cells. Data are shown as mean ± SEM (n = 3). The *P*-values were calculated by paired two-tailed t-test. **P* < 0.05 and ***P* < 0.01.

f. qRT-PCR analysis to validate the RNA-seq results in DNMT1-depleted THP-1 cells. Data are shown as mean ± SEM (n = 3). The *P*-values were calculated by paired two-tailed t-test. **P* < 0.05, ***P* < 0.01 and ****P* < 0.001.

Supplementary Fig 6

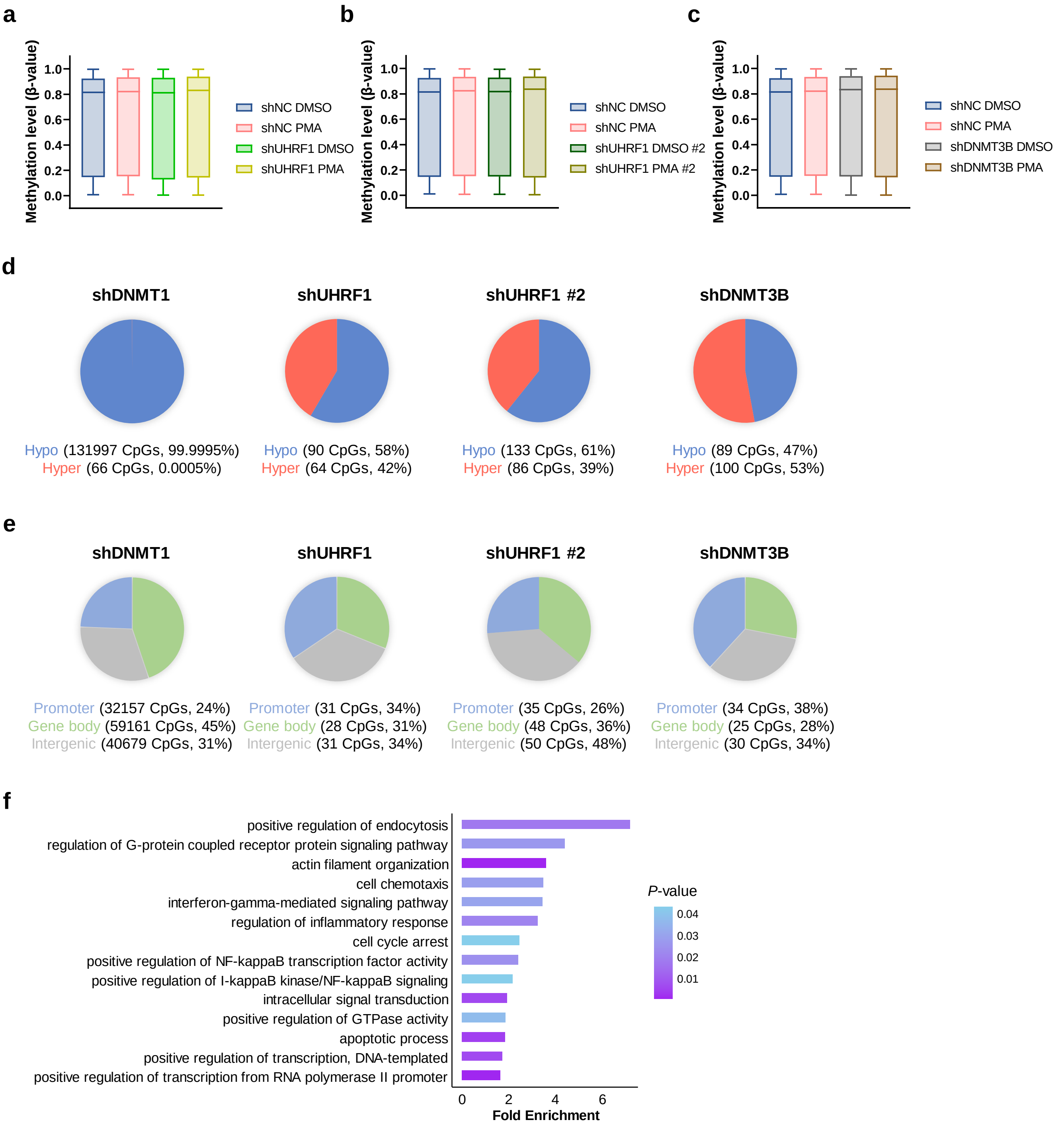

Supplementary Fig 6. Knockdown of UHRF1, DNMT1 and DNMT3B each induces changes in DNA methylation pattern in THP-1 cells.

a. Box plot representing methylation level after BMIQ normalization of each sample. The minimum value, maximum value, and lower and upper quartiles were used for plotting. The line in the middle of each bar indicates the median value.

Supplementary Fig 6

b. Box plot representing methylation level after BMIQ normalization of each sample. The minimum value, maximum value, and lower and upper quartiles were used for plotting. The line in the middle of each bar indicates the median value.

c. Box plot representing methylation level after BMIQ normalization of each sample. The minimum value, maximum value, and lower and upper quartiles were used for plotting. The line in the middle of each bar indicates the median value.

d. Pie chart representing the proportion of hypo- or hyper-methylated CpG islands after the knockdown of each protein. The specific number of CpG islands is indicated.

e. Pie chart representing the regions of hypomethylated CpG islands in each case. The specific number of CpG islands is indicated.

f. GO analysis result with genes related to hypomethylated promoters in DNMT1 knockdown THP-1 cells. Genes associated with the top 600 promoters by log fold change were used as input for GO analysis. The color of the bar represents the *P*-value for each GO term. The x-axis indicates the fold enrichment of each GO term.

Supplementary Fig 7

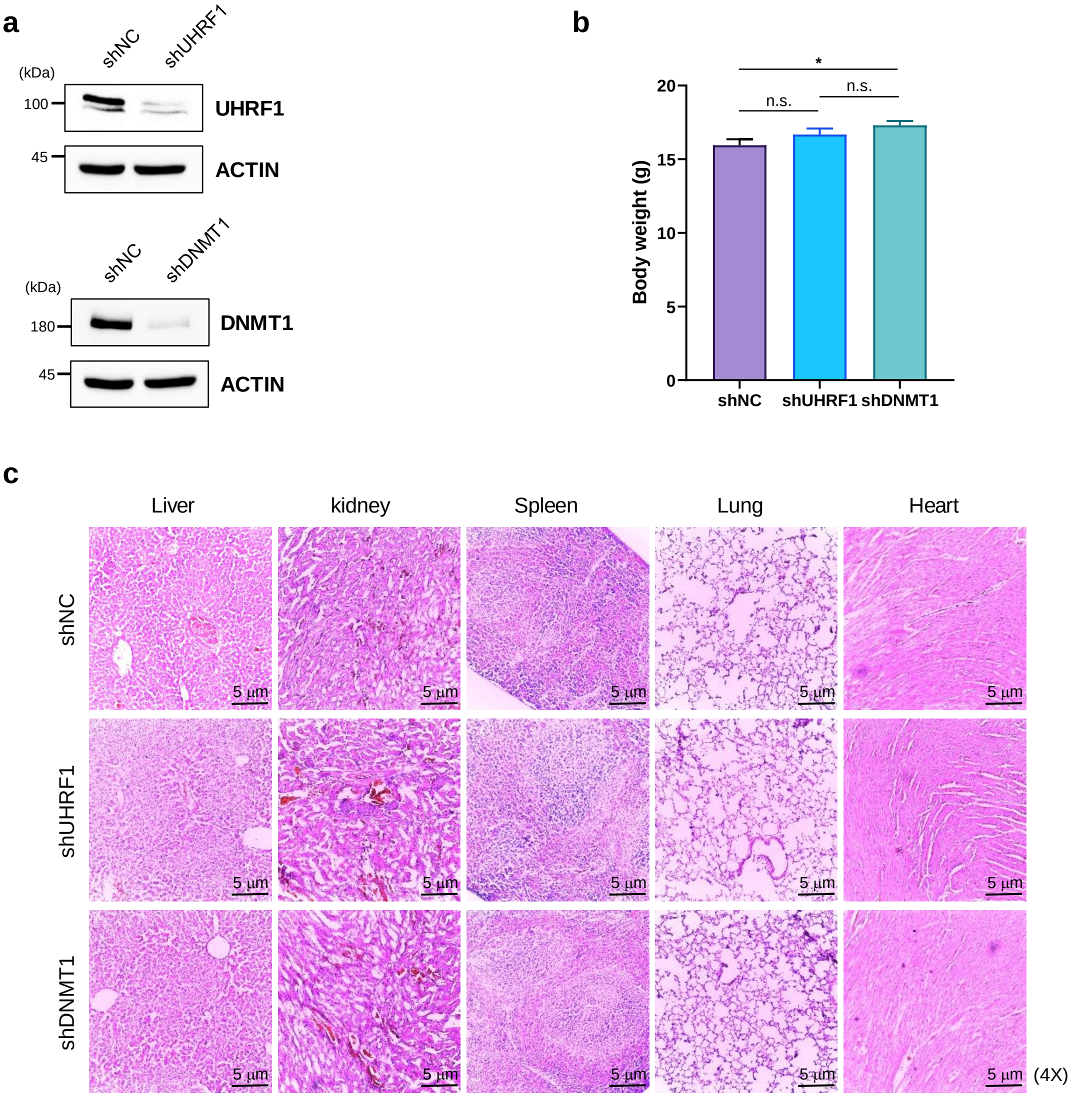

Supplementary Fig 7. Xenotransplantation of different THP-1 cell lines has no effect on body weight and major organ histology.

a. Knockdown validation of each protein from stable cell lines with western blotting before injection. THP-1 cells stably expressing mCherry were used.

b. Body weight of mouse was measured after sacrifice. Data are shown as mean ± SEM (n = 9). The *P*-values were calculated by one-way ANOVA followed by Tukey's multiple comparisons test. *$P < 0.05$ and n.s., not significant.

c. Histology of mouse liver, kidney, spleen, lung and heart tissues of control, UHRF1- or DNMT1-depleted groups. Representative H&E (Hematoxylin and Eosin)-stained histology of mouse liver, kidney, spleen, lung and heart in each group were shown. Scale bar = 5 μm.

Supplementary Fig 8

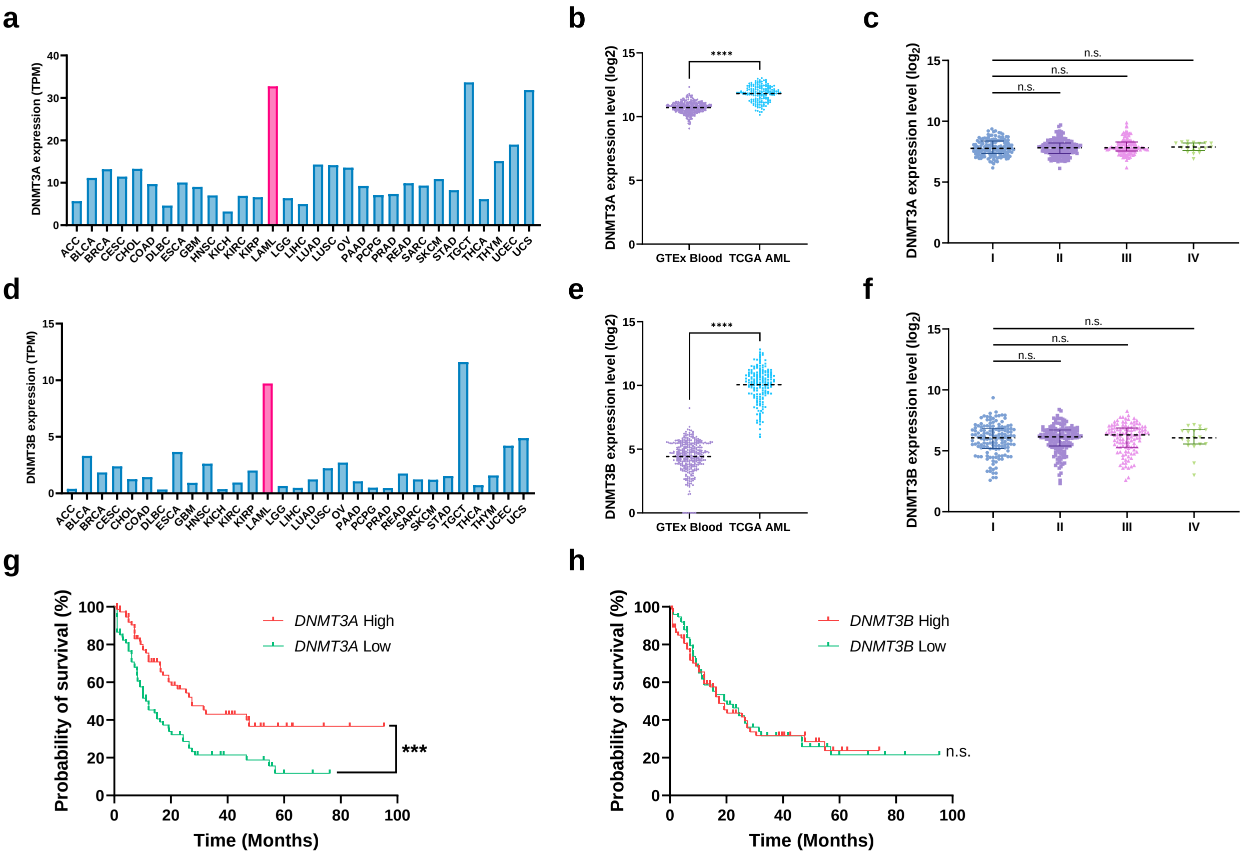

Supplementary Fig 8. Expression level of DNMT3A and DNMT3B are not related to poor prognosis of AML patients.

a. Bar graph showing the relative expression of DNMT3A in 31 different cancer types by GEPIA2 (Gene Expression Profiling Interactive Analysis). The heights of the bars represent the median expression for certain tumor types.

b. Scatter dot plot comparing the relative expression level of DNMT3A in the GTEx blood (n = 337) and TCGA AML datasets (n = 173). This analysis was performed using the UCSC Xena browser. The y-axis shows the $\log_2$ value of the normalized RSEM count. The *P*-value was determined using a two-tailed t-test. ****P < 0.0001.

c. Analysis of DNMT3A expression patterns in blood cancer stages (I [n = 139], II [n = 155], III [n = 113], and IV [n = 19]) in the GENT2 dataset. The y-axis shows the $\log_2$ value of relative mRNA expression levels. The *P*-values were determined using one-way ANOVA followed by Dunnett's multiple comparisons test. n.s., not significant.

d. Bar graph showing the relative expression of DNMT3B in 31 different cancer types by GEPIA2.

Supplementary Fig 8

e. Scatter dot plot comparing the relative expression level of DNMT3B in the GTEx blood (n = 337) and TCGA AML datasets (n = 173). The *P*-value was determined using a two-tailed t-test. ****$P < 0.0001$.

f. Analysis of DNMT3B expression patterns in blood cancer stages (I [n = 139], II [n = 155], III [n = 113], and IV [n = 19]) in the GENT2 dataset. The *P*-values were determined using one-way ANOVA followed by Dunnett's multiple comparisons test. n.s., not significant.

g. Survival analysis of AML patients by OncoLnc. A total of 150 patients were divided into two groups (DNMT3A high [n = 75] and DNMT3A low [n = 75]) based on their expression level. The *P*-value was calculated by log-rank (Mantel–Cox) test. ***$P < 0.001$.

h. Survival analysis of AML patients by OncoLnc. A total of 150 patients were divided into two groups (DNMT3B high [n = 75] and DNMT3B low [n = 75]) based on their expression level. The *P*-value was calculated by log-rank (Mantel–Cox) test. n.s., not significant.

Supplementary Table 1. List of shRNA target sequences used in this study.

Target gene	Sequence (5' to 3')
UHRF1	AGATATAACGTTAGGGTTT
DNMT1	GCCCAATGAGACTGACATCAA
DNMT3B	CCTGTCATTGTTTGATGGCAT

Supplementary Table 2. List of qRT-PCR primer sets used in this study.

Target gene	Direction	Primer (5' to 3')
UHRF1	Forward	AGCCTACTCCCTAGTCCTGG
	Reverse	CCCTGTTGGTGTTGGTGAGT
DNMT1	Forward	GATCTCCTACAACGGGGAGC
	Reverse	AGCCACCAATGCACTCATGT
DNMT3A	Forward	CGAGTCCAACCCTGTGATGAT
	Reverse	CTATCCTGCCATGCTCCAGA
DNMT3B	Forward	TTGGCGACAAGAGGGACATC
	Reverse	ATGCTATCACGGGCCTGTTC
CD14	Forward	AGAACCTTGTGAGCTGGACG
	Reverse	AGTTCCTTGAGGCGGGAGTA
ITGAM (CD11B)	Forward	CGATTCCGTGTTCACCCTGC
	Reverse	TGCCGCTTGAAGAAGCCGAG
PDK4	Forward	GGTGGTGTTCCCCTGAGAAT
	Reverse	GGCAAGCCGTAACCAAAACC
DNASE2	Forward	CCACGTTAGCCAAGAACCCT
	Reverse	GGGCAGGATGCCTACAGTTT
OAS2	Forward	CTGAGCCAGTTGCAGAAAACC
	Reverse	GTCTGCATTGTCGGCACTTTC
PSAT1	Forward	GTCCAGTGGAGCCCCAAAA
	Reverse	TGCCTCCCACAGACCTATGC
TNF	Forward	CTCTTCTGCCTGCTGCACTTTG
	Reverse	ATGGGCTACAGGCTTGTCACTC
IL1B	Forward	CCACAGACCTTCCAGGAGAATG
	Reverse	GTGCAGTTCAGTGATCGTACAGG
IL6	Forward	AGACAGCCACTCACCTCTTCAG
	Reverse	TTCTGCCAGTGCCTCTTTGCTG
GAPDH	Forward	TGCACCACCAACTGCTTAGC
	Reverse	GGCATGGACTGTGGTCATCAG

Full images for western blots

Fig. 1d

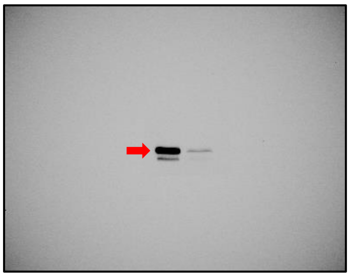

IB: UHRF1

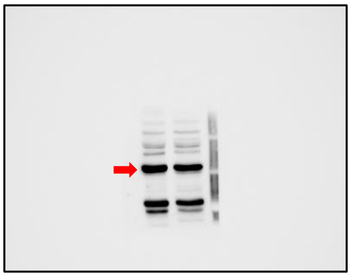

IB: GAPDH

Fig. 1e

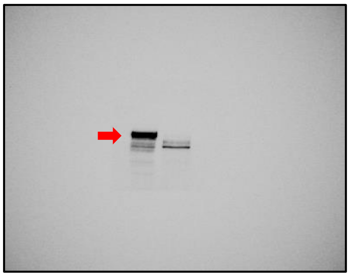

IB: DNMT1

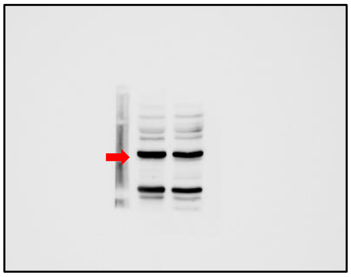

IB: GAPDH

Fig. 1f

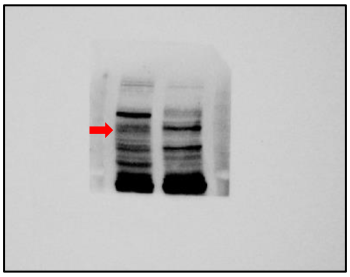

IB: DNMT3A

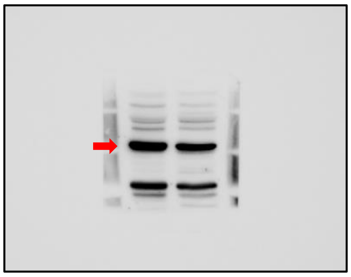

IB: GAPDH

Fig. 1g

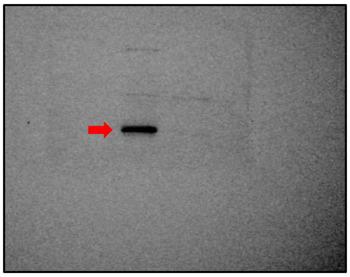

IB: DNMT3B

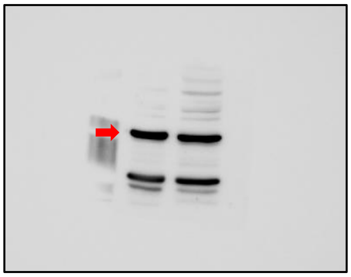

IB: GAPDH

Full images for western blots

Supplementary Fig. 2a

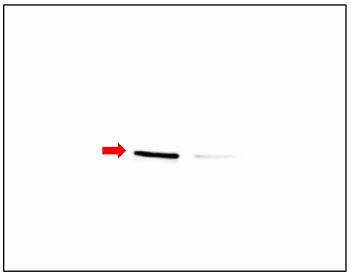

IB: UHRF1

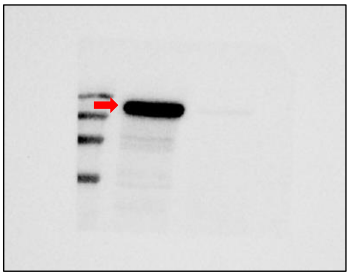

IB: DNMT1

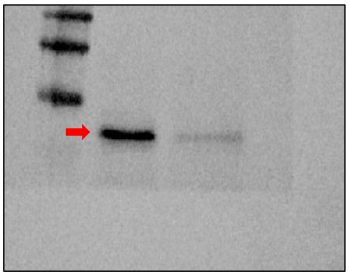

IB: DNMT3B

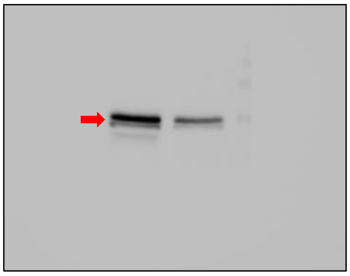

IB: UHRF1 (low contrast)

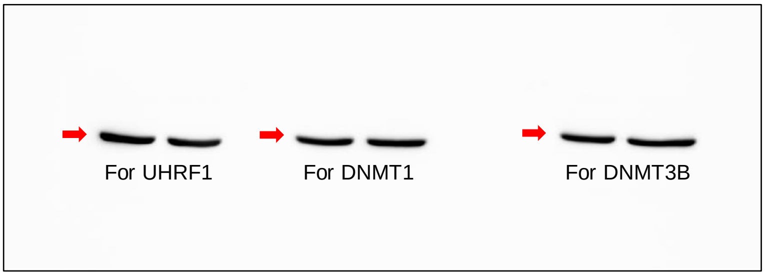

IB: ACTIN

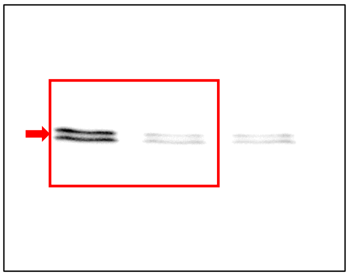

IB: UHRF1 #2

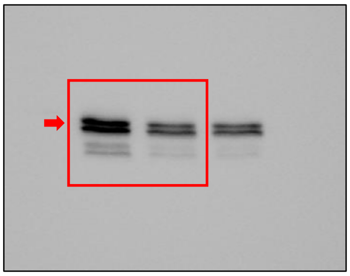

IB: UHRF1 (low contrast)

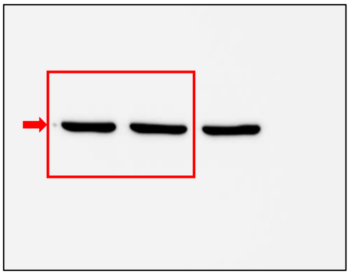

IB: ACTIN

Full images for western blots

Supplementary Fig. 3a

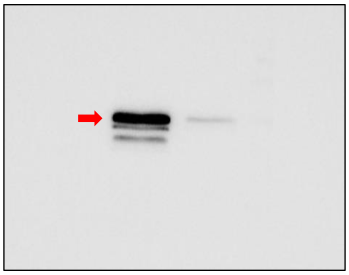

IB: UHRF1

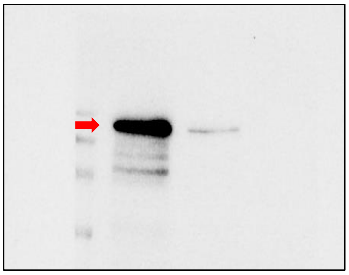

IB: DNMT1

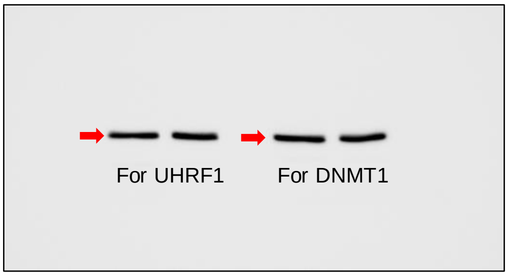

IB: GAPDH

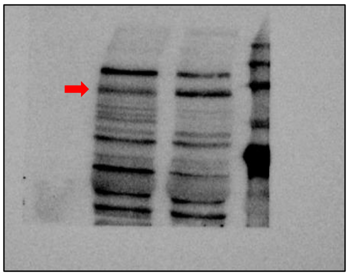

IB: DNMT3A

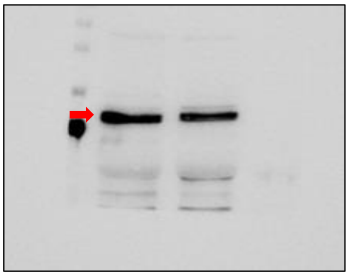

IB: DNMT3B

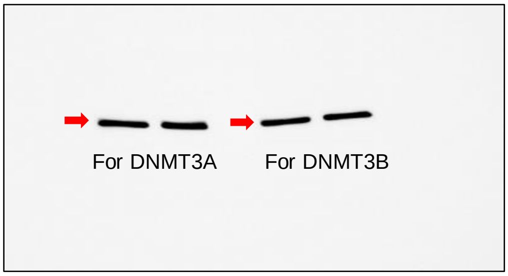

IB: GAPDH

Full images for western blots

Supplementary Fig. 3b

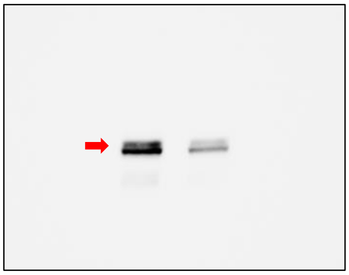

IB: UHRF1

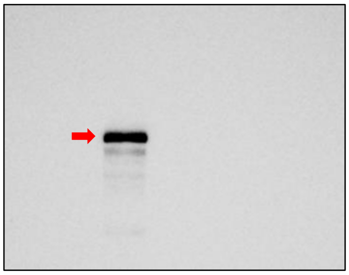

IB: DNMT1

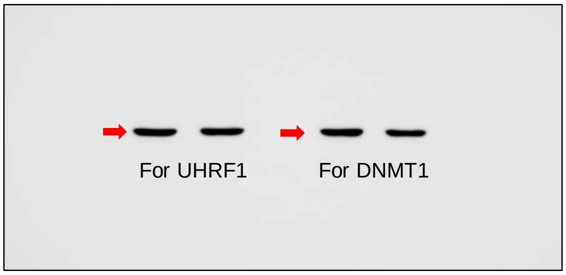

IB: ACTIN

Supplementary Fig. 7a

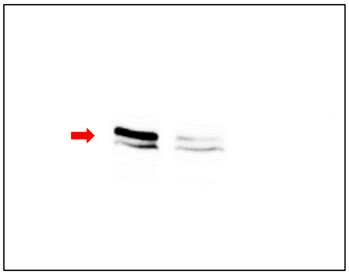

IB: UHRF1

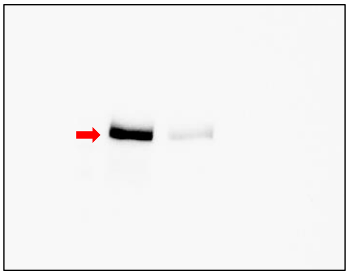

IB: DNMT1

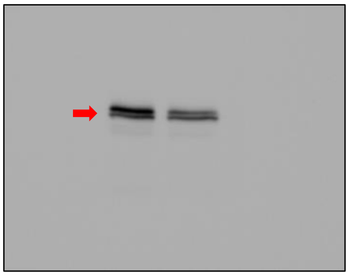

IB: UHRF1 (low contrast)

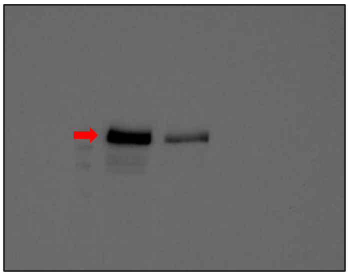

IB: DNMT1 (low contrast)

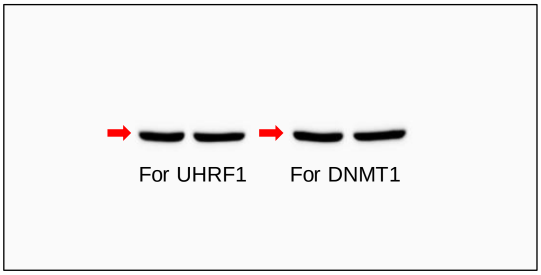

IB: ACTIN
